# Hyperglycaemia and risk of adverse perinatal outcomes: systematic review and meta-analysis

**DOI:** 10.1136/bmj.i4694

**Published:** 2016-09-13

**Authors:** Diane Farrar, Mark Simmonds, Maria Bryant, Trevor A Sheldon, Derek Tuffnell, Su Golder, Fidelma Dunne, Debbie A Lawlor

**Affiliations:** 1 Bradford Institute for Health Research, Bradford Royal Infirmary, Bradford BD9 6RJ, UK; 2 Department of Health Sciences, University of York, York YO10 5DD, UK; 3 Centre for Reviews and Dissemination, University of York, York YO10 5DD, UK; 4 Leeds Institute of Clinical Trials Research, University of Leeds, Leeds LS2 9JT, UK; 5 Hull York Medical School, University of York, York YO10 5DD, UK; 6 Bradford Women’s and Newborn Unit, Bradford BD9 6RJ, UK; 7 Galway Diabetes Research Centre (GDRC) and School of Medicine, National University of Ireland*,* Republic of Ireland; 8 MRC Integrative Epidemiology Unit at the University of Bristol, Oakfield House, Bristol BS8 2BN, UK; 9 School of Social and Community Medicine, University of Bristol, Bristol BS8 2PS, UK

## Abstract

**Objectives** To assess the association between maternal glucose concentrations and adverse perinatal outcomes in women without gestational or existing diabetes and to determine whether clear thresholds for identifying women at risk of perinatal outcomes can be identified.

**Design** Systematic review and meta-analysis of prospective cohort studies and control arms of randomised trials.

**Data sources** Databases including Medline and Embase were searched up to October 2014 and combined with individual participant data from two additional birth cohorts.

**Eligibility criteria for selecting studies** Studies including pregnant women with oral glucose tolerance (OGTT) or challenge (OGCT) test results, with data on at least one adverse perinatal outcome.

**Appraisal and data extraction** Glucose test results were extracted for OGCT (50 g) and OGTT (75 g and 100 g) at fasting and one and two hour post-load timings. Data were extracted on induction of labour; caesarean and instrumental delivery; pregnancy induced hypertension; pre-eclampsia; macrosomia; large for gestational age; preterm birth; birth injury; and neonatal hypoglycaemia. Risk of bias was assessed with a modified version of the critical appraisal skills programme and quality in prognostic studies tools.

**Results** 25 reports from 23 published studies and two individual participant data cohorts were included, with up to 207 172 women (numbers varied by the test and outcome analysed in the meta-analyses). Overall most studies were judged as having a low risk of bias. There were positive linear associations with caesarean section, induction of labour, large for gestational age, macrosomia, and shoulder dystocia for all glucose exposures across the distribution of glucose concentrations. There was no clear evidence of a threshold effect. In general, associations were stronger for fasting concentration than for post-load concentration. For example, the odds ratios for large for gestational age per 1 mmol/L increase of fasting and two hour post-load glucose concentrations (after a 75 g OGTT) were 2.15 (95% confidence interval 1.60 to 2.91) and 1.20 (1.13 to 1.28), respectively. Heterogeneity was low between studies in all analyses.

**Conclusions** This review and meta-analysis identified a large number of studies in various countries. There was a graded linear association between fasting and post-load glucose concentration across the whole glucose distribution and most adverse perinatal outcomes in women without pre-existing or gestational diabetes. The lack of a clear threshold at which risk increases means that decisions regarding thresholds for diagnosing gestational diabetes are somewhat arbitrary. Research should now investigate the clinical and cost-effectiveness of applying different glucose thresholds for diagnosis of gestational diabetes on perinatal and longer term outcomes.

**Systematic review registration** PROSPERO CRD42013004608

## Introduction

Gestational diabetes, defined as hyperglycaemia first identified during pregnancy, increases the risk of a range of adverse perinatal outcomes including macrosomia and caesarean section.^1^ There is also growing evidence that the longer term health of the mother and infant could be adversely affected.^2^ ^3^ ^4^ The primary aim of diagnosing gestational diabetes is to identify women and infants at risk of short or longer term adverse outcomes. While traditionally the primary aim was to identify women at risk of type 2 diabetes, the recent International Association of Diabetes and Pregnancy Study Groups (IADPSG) proposed glucose thresholds were calculated to identify adverse perinatal outcomes with the ultimate aim of preventing future obesity in offspring.^5^

Although treatment of gestational diabetes can reduce the risk of perinatal outcomes,^6^ ^7^ there is uncertainty regarding the optimal glucose threshold (at oral glucose tolerance testing (OGTT)) that should define gestational diabetes. Findings from the Hyperglycaemia and Adverse Pregnancy Outcomes (HAPO) study showed graded linear increases in large for gestational age, large skinfold thicknesses, high cord blood C peptide, and several other important perinatal outcomes, across the whole distribution of fasting and post-load glucose in women without existing diabetes or gestational diabetes.^8^ Given the lack of any clear threshold for increased risk, the IADPSG calculated thresholds using the HAPO data as the glucose values at which odds for birthweight, cord C peptide, and percent body fat above the 90th centile reached 1.75 times the estimated odds of these outcomes above mean glucose values.^5^ The IADPSG criteria for diagnosing gestational diabetes have been endorsed by the World Health Organization (WHO)^9^ and more recently by the International Federation of Gynecology and Obstetrics (FIGO).^10^ Not all countries or institutions, such as UK National Institute of Health and Care Excellence (NICE)^11^ and American College of Obstetrics and Gynaecology,^12^ have endorsed these criteria. Though the HAPO study is large, multicentred, and well conducted, it did not present results by country, and the shape and magnitude of the association between glycaemia and pregnancy outcomes could differ in different populations, for example by ethnicity.

The question of whether the shape and magnitude of the association would be seen in all populations remains unanswered. We recently analysed a cohort of white British and south Asian women^13^ and found that the HAPO/IADPSG findings were replicated in the white British women, but in the south Asian women our results suggested that lower fasting and post-load glucose concentrations are required to achieve the same odds of identifying adverse perinatal outcomes. We also noted that the IADPSG thresholds for post-load glucose were importantly influenced by the fact that the post-load threshold used by HAPO to exclude women with gestational diabetes was much higher than that currently used in clinical practice and also at the time of the start of that study. A further issue is whether using a different set of outcomes would produce different diagnostic thresholds to those selected by the IADPSG, as, even with linear relations, the slopes are likely to differ and hence the threshold at which a given odds ratio would occur will differ between outcomes. In particular the IADPSG did not consider important clinical outcomes such as hypertensive disorders of pregnancy, the requirement for induction of labour, caesarean section, and whether the infant suffered from shoulder dystocia, neonatal hypoglycaemia, and/or required admission to neonatal intensive care, which are key clinical criteria that clinicians and pregnant women are concerned about.

We conducted a systematic search of the literature to examine the available evidence and the degree to which these questions have been examined in different populations. Wherever possible we pooled data and conducted appropriate sensitivity analyses to investigate any potential study and population effects.

## Methods

We conducted this systematic review and meta-analysis in accordance with Cochrane Systematic Reviews^14^ and the Centre for Reviews and Dissemination recommendations^15^ and have reported our findings according to PRISMA reporting guidelines.

### Search strategy

Searches were undertaken, and three reviewers (DF, MS and SG) independently assessed the literature for inclusion. Data from eligible studies were combined with data from two additional birth cohort studies: the Born in Bradford cohort^13^ and the Atlantic Diabetes in Pregnancy cohort (F Dunne, personal communication), for which we also had access to individual participant data.

#### Identification of studies from systematic review

We searched the literature in September 2013 and October 2014 using Medline and Medline in-Process, Embase, CINAHL Plus, the Cochrane Central Register of Controlled Trials (CENTRAL), Cochrane Database of Systematic Reviews (CDSR), Database of Abstracts of Reviews of Effects (DARE), Health Technology Assessment database (HTA), NHS Economic Evaluation Database (NHS EED), and Cochrane Methodology Register (CMR). The full Medline search strategy is shown in the appendix 1 and was appropriately translated for the other databases.

#### Identification of studies from unpublished individual participant data

We had access to three cohort studies with individual participant data: Born in Bradford (BiB; J Wright, personal communication); Atlantic Diabetes in Pregnancy (Atlantic-DIP; F Dunne, personal communication); and the Warwick/Coventry cohort (P Saravanan, personal communication). The Warwick/Coventry cohort had insufficient complete case data and was not included.

Born in Bradford is a prospective birth cohort; the study methods have been previously described.^16^ All women booked for delivery in Bradford are offered a 75 g oral glucose tolerance test (OGTT) at around 26-28 weeks’ gestation, and women were recruited mainly at their OGTT appointment.^13^ The Atlantic DIP is a multicentre cohort study comprising a partnership of five hospitals at the Irish Atlantic seaboard. It was set up in 2006 with a focus on research, audit, clinical care, and professional and patient education for diabetes in pregnancy.^17^ As with the Bradford study, women were offered a 75 g OGTT at 24-28 weeks’ gestation from September 2006 to April 2012. Data on women with singleton pregnancies were collected from study entry until 12 weeks’ postpartum.^18^

### Study selection: inclusion and exclusion criteria

To be eligible, studies had to include pregnant women who had undergone an OGTT (comprising fasting and one, two, and three hour post-load samples) or oral glucose challenge test (OGCT) (comprising a non-fasting and one hour post-load sample) with measures of fasting and/or post-load glucose concentration. We excluded women from our analyses if they had pre-existing diabetes or had a diagnosis of gestational diabetes (using various criteria thresholds set by each included study; see table 1 for criteria and tables 2-4[Table tbl2 tbl3 tbl4] for glucose thresholds) because they would have received treatment and this would have influenced the natural association between glucose and outcome. Studies had to provide data on at least one perinatal adverse outcome in a form that could be included in the meta-analyses (number of women and events in each glucose category).

**Table 1 tbl1:** Recommended criteria for diagnosis of gestational diabetes with oral glucose tolerance test (OGTT)

	Fasting	One hour post-load	Two hour post-load	Three hour post-load
**75 g OGTT (plasma glucose)**				
IADPSG^5^ (2010), ADIPS (2013), WHO^9^ (2013)*	≥5.1	≥10.0	≥8.5	—
WHO^19^ (1999)*	≥6.1	—	≥7.8	—
ADA^20^ (2006)*	≥5.3	≥10.0	≥8.6	—
ADIPS^21^ (1998)*	≥5.5	—	≥8.0	—
**100 g OGTT (plasma or serum glucose)**				
ACOG^12^/C-C†	≥5.3	≥10.0	≥8.6	≥7.8
NDDG^22^†	≥5.8	≥10.6	≥9.2	≥8.0
O’Sullivan^23^†	≥5.0	≥9.2	≥8.1	≥6.9

IADPSG=International Association of Diabetes and Pregnancy Study Groups; ACOG=American College of Obstetricians and Gynecologists; ADIPS=Australasian Diabetes in Pregnancy Society; ADA=American Diabetes Association; C&C=Carpenter & Coustan criteria^24^; NDDG=National Diabetes Data Group; WHO=World Health Organization.

*One threshold should be equalled or exceeded for diagnosis of gestational diabetes.

†Two thresholds should be equalled or exceeded for diagnosis of gestational diabetes.

**Table 2 tbl2:** Characteristics of included studies that used 50 g oral glucose challenge test (OGCT) or 100 g oral glucose tolerance test (OGTT) for diagnosis of gestational diabetes

	No of women	Test type	Test timing*			Diagnosis exclusion criteria (mmol/L)	Outcomes†								
			F	1	2		LGA	Macrosomia	S dystocia	Neonatal hypoglyc	Pre-eclampsia/PIH	Preterm birth	C section	Ind labour	Ins delivery
Carr,^25^ 2011, US (Seattle)	25 969	50 g OGCT	—	X	—	100 g OGTT ≥2 values fasting ≥5.3, 1 hour ≥10.0, 2 hour ≥8.6, and 3 hour ≥7.8	—	—	—	—	X	X	—	—	—
Chandna,^26^ 2006, Pakistan (Karachi)	633	50 g OGCT	—	X	—	NR	—	—	—	X	X	—	X	—	X
Cheng,^27^ 2007, US (California)	13 901	50 g OGCT	—	X	—	NR	X	X	X	X	—	—	—	X	—
Figueroa,^28^ 2013, US (multicentre)	1839	50 g OGCT	—	X	—	100 g OGTT fasting <5.3 + ≥2 values: 1 hour ≥10.0, 2 hour ≥8.6, 3 hour ≥7.8	X	X	—	X	—	—	—	—	—
Hillier,^29^ 2008, US (Hawaii and Portland)	41 450	50 g OGCT	—	X	—	100 g OGTT Two criteria used: (i) ≥2 values: fasting ≥5.8 or 1 hour ≥10.5 or 2 hour ≥9.2 or 3 hour ≥8.0; (ii) ≥2 values: fasting ≥5.3 or 1 hour ≥10.0 or 2 hour ≥8.6 or 3 hour ≥7.8	—	X	—	—	—	—	—	—	—
Ong,^30^ 2008, UK (Cambridge)	3826	50 g OGCT	—	X	—	50 g OGCT 1-hr >7.8 and 75 g OGTT levels NR. Fasting >6.1 2 hour level NR	—	—	—	—	—	—	X	—	X
Scholl,^31^ 2001, US (New Jersey)	1157	50 g OGCT	—	X	—	NR	X	—	—	—	X	X	X	—	—
Sermer,^32^ 1995, Canada (Toronto)	3637	50 g OGCT / 100 g OGTT	X	X	X	100 g OGTT ≥2 values: fasting >5.8 or 1 hour >10.5 or 2 hour >9.1 or 3 hour >8.0	—	X	—	—	X	—	X	—	—
Witter,^33^ 1988, US (Baltimore)	3897	50 g OGCT	—	X	—	100 g OGTT ≥2 values: fasting ≥5.8 or 1 hour ≥10.5 or 2 hour >9.1 or 3 hour >8.0	—	X	—	—	—	—	—	—	—
Yee,^34^ 2011, US (California)	13 789	50 g OGCT	—	X	—	100 g OGTT ≥2 values: fasting >5.8 or 1 hour ≥10.5 or 2 hour ≥9.1 or 3 hour ≥8.0	X	X	X	—	X	—	X	—	—

LGA=large for gestational age; NR=not reported.

*F=fasting, 1=one hour post-load, 2=two hours post-load.

†Large for gestational age, macrosomia, shoulder dystocia, neonatal hypoglycaemia, pre-eclampsia/PIH (pregnancy induced hypertension), preterm birth, caesarean section, induced labour, instrumental delivery.

**Table 3 tbl3:** Characteristics of included studies that used 75 g oral glucose tolerance test (OGTT) for diagnosis of gestational diabetes

	No of women	Test timing*			Diagnosis exclusion criteria (mmol/L)	Outcomes†								
		F	1	2		LGA	Macrosomia	Sh dystocia	Neonatal hypogly	Pre-eclampsia/PIH	Preterm birth	C section	Ind labour	Ins delivery
Aris,^35^ 2014, Singapore	1081	X	—	X	75 g OGTT fasting ≥7.0 or 2 hour ≥7.8	X	—	—	—	—	—	—	—	—
Atlantic Dip, 2015, Ireland (west coast)	4869	X	—	X	75 g OGTT fasting ≥6.1 or 2 hour ≥7.8	X	X	X	—	X	X	X	—	X
BIB, 2015, UK (Bradford)	9645	X	—	X	75 g OGTT fasting ≥6.1 or 2 hour ≥7.8	X	X	X	—	X	X	X	X	X
HAPO group,^8^ 2008, international multicentre	23 316	X	X	X	75 g OGTT fasting > 5.8 or 2 hour >11.1 or RPG >8.9	X	—	—	X	—	—	X	—	—
HAPO group,^36^ 2010, international multicentre	21 364	X	X	X	75 g OGTT fasting >5.8 or 2 hour >11.1 or RPG >8.9	—	—	—	—	X	—	—	—	—
Jensen,^37^ 2001, Denmark (multicentre)	2904	X	—	X	75 g OGTT ≥2 values: fasting >5.7 or 30 mins >11.9 or 1 hour 12.0 or 90 mins >9.7 or 2 hour >8.9 or 180 mins >7.4	X	X	X	X	X	X	X	X	X
Kerenyi,^38^ 2009, Hungary (Budapest)	3787	X	—	X	75 g OGTT fasting ≥7.0 or 2 hour ≥7.8	X	—	—	—	—	—	—	—	—
Lao,^39^ 2003, China (Hong Kong),	2168	—	—	X	75 g OGTT 2 hour ≥8.0	X	X	—	—	—	X	X	—	—
Metzger,^40^ [HAPO] 2010 International multicentre	17 094	X	X	X	75 g OGTT fasting >5.8 or 2 hour >11.1 or RPG >8.9	—	—	—	X	—	—	—	—	—
Moses,^41^ 1995, Australia (Illawarra, NSW)	1441	—	—	X	75g OGTT 2 hour ≥8.0	X	—	—	—	—	—	X	—	X
Pettitt,^42^ 1980, US (Arizona)	811	—	—	X	75g OGTT 2 hour ≥11.1	X	—	—	—	—	X	X	—	—
Savona-Ventura,^43^ 2010, Malta	1289	X	—	X	NR	—	X	—	—	X	—	—	—	—

NR=not reported; RPG=random plasma glucose.

*F=fasting, 1=one hour post-load, 2=two hours post-load.

†Large for gestational age, macrosomia, shoulder dystocia, neonatal hypoglycaemia, pre-eclampsia/PIH (pregnancy induced hypertension), preterm birth, caesarean section, induced labour, instrumental delivery.

**Table 4 tbl4:** Characteristics of included studies using 50 g oral glucose challenge test (OGCT) or 100 g oral glucose tolerance test (OGTT) for diagnosis of gestational diabetes

	No of women	Test type	Test timing*			Diagnosis exclusion criteria (mmol/L)	Outcomes†								
			F	1	2		LGA	Macrosomia	Sh dystocia	Neonatal hypogly	Pre-eclampsia/PIH	Preterm birth	C section	Ind labour	Ins delivery
Landon,^44^2011, US (multicentre)	1368	100 g OGTT	X	X	X	Fasting >5.3	X	—	X	—	X	—	—	—	—
Little,^45^1990, US (Missouri)	287	100 g OGTT	—	—	X	75 g OGTT fasting >5.7 or 2 hour >9.2	X	—	X	X	—	—	X	—	—
Riskin-Mashiah,^46^ 2009, Israel (Haifa)	6129	100 g OGTT	X	—	—	100 g OGTT first trimester fasting >5.8	—	X	—	—	—	—	X	—	—
Sermer,^32^ 1995, Canada (Toronto)	3637	50 g OGCT/100 g OGTT	X	X	X	100 g OGTT ≥2 values: fasting >5.8 or 1 hour >10.5 or 2 hour >9.1 or 3 hour >8.0	—	X	—	—	X	—	X	—	—
Tallarigo,^47^ 1986, Italy (Pisa)	249	100 g OGTT	—	—	X	100 g OGTT ≥2 values: fasting >5.8 or 1 hour >10.5 or 2 hour >9.1 or 3 hour >8.0	—	X	—	—	—	X	X	—	—

*F=fasting, 1=one hour post-load, 2=two hours post-load.

†Large for gestational age, macrosomia, shoulder dystocia, neonatal hypoglycaemia, pre-eclampsia/PIH (pregnancy induced hypertension), preterm birth, caesarean section, induced labour, instrumental delivery.

### Data extraction and quality assessment

Two reviewers (MS and SG) extracted data and conducted the quality assessments. Any disagreements between reviewers were resolved through discussion, including with other authors as necessary. Risk of bias in the included studies was assessed with a modified version of the Critical Appraisal Skills Programme (CASP) and Quality in Prognostic Studies (QUIPS) assessment tools, designed for observational studies of association and prediction.^48^ When undertaking quality assessment of the studies, we considered the representative nature of the included population; loss to follow-up; consistency of glucose measurement and outcome assessment; blinding of participants and medical practitioners to glucose concentration; blinding of outcome assessors to glucose concentration; and selective reporting of outcomes. We also extracted information on any adjustment for covariates, though our interest here is on a diagnostic threshold of glucose and in clinical practice this would not be adjusted for. We therefore aimed primarily to use unadjusted associations. Each criterion was classified as at low, high, or unclear risk of bias.

All of the studies reported numbers of women and numbers of adverse outcomes in a range of glucose categories. Data on these glucose categories (such as range and/or median concentration for each category, numbers of women and of outcomes in each category) were extracted for OGTT (75 g and 100 g test (fasting and one hour and two hour post-load)) and one hour 50 g OGCT. Data were extracted for the following perinatal outcomes: induction of labour; caesarean section (elective or emergency); instrumental delivery (ventouse or forceps); pregnancy induced hypertension (pre-eclampsia; macrosomia (birthweight ≥4000 g); large for gestational age (≥90th birthweight centile); preterm birth (<37 weeks’ gestation); birth injury/trauma (shoulder dystocia, Erb’s palsy, fractured clavicle), and neonatal hypoglycaemia. We also extracted sociodemographic and clinical data, such as age range of participants, how those with diabetes were excluded, and parity.

For the two studies with individual participant data we created seven categories of glucose concentration for both fasting and two hour post-load glucose concentrations, designed to include approximately equal numbers of women in each category. The numbers of women and numbers of adverse outcomes in each category were then calculated for each outcome to generate summary data similar to that extracted from publications.

### Statistical analysis

Analyses were based on the number of women and number of adverse perinatal outcomes in each glucose category in each study. Use of these raw numbers means that we did not adjust our results for any covariates. Our aim, however, was to determine whether there were clear glucose thresholds for diagnosis of gestational diabetes across a range of pregnancy and perinatal outcomes and not to assess causality. Thus, confounding is not a concern and reflects clinical practice (where glucose thresholds without adjustment are used) and the lack of any adjustment for covariates is therefore appropriate here. We explored whether results were heterogeneous and, if so, whether this related to characteristics that differ between participants in the different studies, which was relevant to our aim of determining whether the HAPO/IADPSG results were generalisable.

One study^44^ presented only adjusted odds ratios (adjusted for maternal age, gestational age at enrolment and at delivery, parity, BMI, and race or ethnicity). With the exception of that one study all other results from all other studies were the unadjusted associations that we required.

To determine whether any glucose threshold exists above which women or infants are at significantly greater risk of adverse perinatal outcomes, we tested the validity of the assumption of a log-linear association between outcome and glucose concentration by visual assessment (based on plotting the results from each study and by using a model with an additional “glucose-squared” term. A significant association with glucose squared would suggest a quadratic-curvilinear association.

After our initial visual assessment of glucose and perinatal outcome plots, we modelled associations across studies in a “one stage” hierarchical logistic regression analysis.^49^ The numbers of women with an outcome event in each glucose category was regressed against the average glucose concentration in each category. Independent intercepts and random effects on the slopes across studies were included to allow the baseline risk and the association between glucose concentration and outcome to vary between studies, thus accounting for any potential heterogeneity. We used mixed effects logistical regression routines in R software for the modelling. We assessed the percentage of variance between study findings not due to chance by determining the I^2^ statistic.^14^ When an outcome was reported in only one study, we fitted the same logistic regression model but without the meta-analysis component to estimate the association between outcome and glucose concentration as for outcomes reported by several studies.

Associations were modelled separately for each outcome, glucose test (75 g OGTT, 100 g OGTT, 50 g OCGT), and timing of the measurement of glucose concentration (fasting and one hour or two hour post-load). These models produced a summary estimate across studies for the association between concentration and outcome in terms of the odds ratio of outcome per 1 mmol/L increase in concentration. Full details of the statistical methods and models are provided in appendix 2.

To increase the number of studies and participants we combined the fasting glucose results from the 75 g and 100 g OGTT in meta-analyses using the logistic regression models described above because fasting glucose should not be affected by the subsequent glucose test load (75 g or 100 g). We also combined the 75 g and 100 g one hour post-load results and the 75 g and 100 g two hour post-load results, assuming the associations between glucose and outcomes were the same for both tests.

We conducted two sensitivity analyses, one that excluded studies with a high or unclear risk of surveillance and detection bias (lacked blinding) from analyses (leaving four published reports related to two studies^8^ ^32^ ^36^ ^50^). We also examined the influence of study population/region of residence on estimates using the 75 g and 100 g OGTT by dividing studies into five categories (international, North America, Europe, Asia, Australasia) and repeating the meta-analyses within each region. These regions were chosen once we had completed our search and are based on identified relevant studies.

### Patient involvement

As this is a systematic review and meta-analyses using conventional methods we did not seek the views of women in the design or conduct of our study. The outcomes we included in this review were those identified by the Cochrane Pregnancy and Childbirth Group (CPCG) as being essential for reviews of diabetes in pregnancy. The CPCG includes relevant patients/service users (in this case women of reproductive age and/or women who have had gestational diabetes) who contribute to decisions about which outcomes are included in the standard list.

## Results

### Details of included and excluded studies

Figure 1 shows the number of reports and studies identified and numbers included and excluded. After screening titles and abstracts, we obtained 125 study reports for full text review. After full text review we included 25 published reports detailing associations between perinatal outcomes and maternal glucose concentrations. At title and abstract screening, studies were excluded mainly because they were not answering the question we were examining. At full text screening, studies were excluded mostly because they did not present data (conference abstracts), did not report any of our included outcomes, did not report outcomes by glucose concentrations, or did not report data in a form that could be included or converted for inclusion in the meta-analyses. Published studies were combined with the two individual participant data cohorts; BiB and Atlantic DIP. Tables 2, 3, and 4[Table tbl2 tbl3 tbl4] summarise the characteristics of the included publications and individual participant data cohorts.

**Figure f1:**
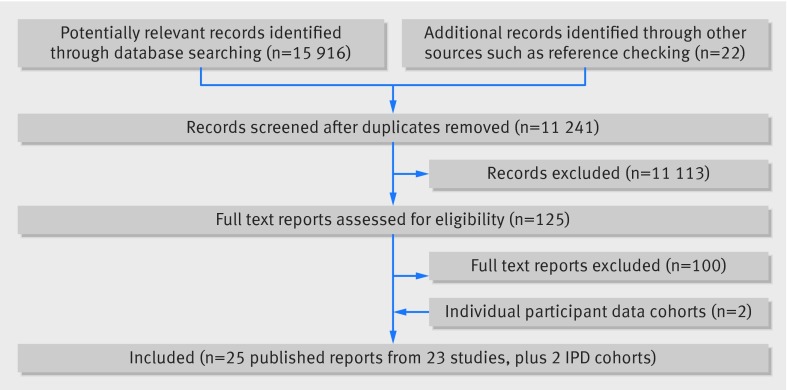
**Fig 1** Flow diagram for selection of studies of hyperglycaemia and risk of adverse perinatal outcomes

### Quality assessment

Generally, studies had a low risk of bias (table A, appendix 3), with the exception of surveillance and detection bias, which was high or unclear for all but four published reports related to two studies. Most studies recruited any pregnant women without pre-existing diabetes or newly diagnosed gestational diabetes, often at the study hospital’s gestational diabetes screening clinic. Few studies applied any further inclusion/exclusion criteria so the study populations’ are likely to be representative of the general obstetric population at the study site. Studies generally did not report comprehensive demographic details of participants and did not report results by subgroups, including by ethnicity. In studies that included a proportion of women with gestational diabetes and reported outcomes separately, we extracted only data for those without gestational diabetes. Most studies were in Western populations from high income countries, with a small number from other populations, such as the Pima Indian population of Arizona. Most studies had minimal loss to follow-up. Studies diagnosed gestational diabetes (and excluded women) with both the one and two step approach with either the 75 g or 100 g OGTT and with various glucose thresholds (tables 3 and 4[Table tbl3 tbl4]).

The main potential risk of bias was due to lack of blinding of glucose concentrations after OGTT. This could have resulted in surveillance or detection bias (and potentially to a self fulfilling prophesy). For example, pregnancy surveillance might have been increased in women with higher glucose concentrations, which could have increased the likelihood of interventions including induction of labour or caesarean section or the scrutiny with which other outcomes are determined, compared with surveillance in women with lower concentrations.

### Linear associations of glucose with perinatal outcomes

Figure 2 shows the pooled results for the association between increases in fasting glucose and results of one hour post-load 50 g OGCT, two hour 75 g OGTT, and two hour 100 g OGTT and each perinatal outcome.

**Figure f2:**
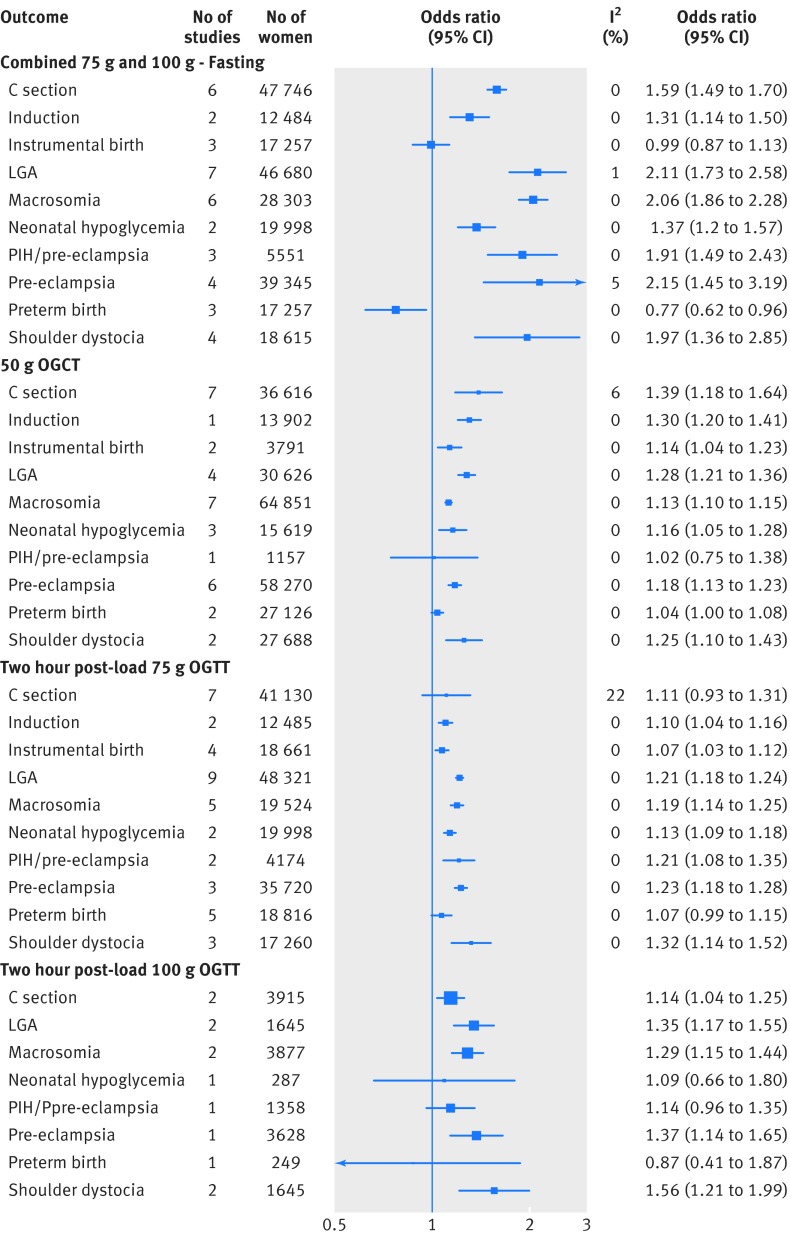
**Fig 2** Odd ratios for outcomes associated with glucose concentration (fasting combined 75 g and 100 g OGTT, one hour post-load 50 g OGCT, two hour post-load 75 g OGTT, and two hour post-load 100 g OGTT)

For all glucose exposures there were positive associations with caesarean section, induction of labour, large for gestational age, macrosomia, and shoulder dystocia. In general for these outcomes, the magnitudes of association were stronger for fasting glucose compared with any of the post-load glucose measurements. For example, the odds ratios for large for gestational age per 1 mmol/L increase of fasting and two hour post-load glucose concentrations (after a 75 g OGTT) were 2.15 (95% confidence interval 1.60 to 2.91) and 1.20 (1.13 to 1.28), respectively (figure C, appendix 3). Increases in fasting glucose concentration was also clearly inversely associated with preterm delivery, whereas the association between post-load concentration and this outcome was more inconsistent: weakly positive for 50 g one hour OGCT, weakly positive for 75 g two hour OGTT, and inverse with 100 g two hour OGTT; but for some of these, particularly the latter, the confidence intervals were wide and included the null. We found that results of 50 g one hour post-load OGCT and 75 g two hour OGTT were positively associated with instrumental delivery, whereas fasting glucose concentration was not clearly associated with this outcome (no studies using a 100 g OGTT reported this outcome). All glucose measurements, except the two hour 100 g post-load glucose concentration from the OGTT, were positively associated with neonatal hypoglycaemia. The 75 g two hour post-load OGTT result was positively associated with combined pregnancy induced hypertension/pre-eclampsia, but there was no consistent association between results of the 50 g OGCT or 100 g two hour post-load OGTT and this outcome.

When we pooled two hour post-load glucose associations with outcomes for studies that used either a 75 g or a 100 g OGTT, the pattern of associations were broadly similar to those when the two sets of studies were considered separately (fig A, appendix 3).

Associations between glucose concentrations and outcomes were generally monotonic, suggesting linear associations across the distribution with no clear threshold at which risk substantially increases (figs A-I, appendix 4). The quadratic statistical tests largely supported the linear association, with some possible flattening of the positive association with pregnancy induced hypertension combined with pre-eclampsia or pre-eclampsia alone at the upper end of the post-load glucose distribution (table B, appendix 3). Few studies assessed one hour post-load glucose concentration for either 75 g or 100 g OGTT, and only a subset of the outcomes were examined in those studies for this exposure. In general results for the one hour post-load concentration were broadly similar to those for the two hour post-load concentration, but, given the limited amount of data for these associations, estimates were less precise with wider confidence intervals.

### Sensitivity and subgroup analyses

Figures K and L in appendix 3 show the pooled results for the association between fasting and two hour post-load glucose concentration (75 g OGTT ) and each perinatal outcome, excluding all results from the two studies (four published reports) that were least likely to have bias because of lack of blinding.

Our analyses were limited by the fact that there were only two studies for which we could ascertain that clinical staff were definitely blinded, one of which was the largest study included in the whole meta-analyses.^8^ Broadly, results for fasting glucose and two hour post-load concentrations were similar between studies with definite blinding and those without blinding or where we were unsure (figs K and L, appendix 3). The association between fasting glucose concentration, but not two hour post-load concentration, and birth size (both large for gestational age and macrosomia), but not other outcomes, seemed stronger for the blinded studies (large for gestational age^8^ and macrosomia^32^) than all other studies pooled together.

Exclusion of studies with blinding left data from only one study examining the association between fasting glucose concentration and neonatal hypoglycaemia. This study included 2904 women and showed a positive association, with point estimates that were higher than those in the main meta-analysis without these exclusions (odds ratio 1.43 (95% confidence interval 0.64 to 3.22) and 1.37 (1.20 to 1.57), respectively).^37^ Because of the small sample size of this one study, however, the confidence intervals were wide and included the null result. The only other study with this outcome at fasting was HAPO, and the results from that study (based on the point estimate) suggested a possible weaker association, but the results from the two studies are consistent with each other (odds ratio 1.37 (1.20 to 1.57) for HAPO alone). Similarly, after exclusions only two studies with 3191 women remained for the two hour post-load association with neonatal hyperglycaemia. The point estimates were the same as the main analyses, though, again because of the reduced sample size, the confidence intervals were wide and included the null result.

We examined the effect of region on the association between fasting concentration and two hour post-load concentration (75 g OGTT) and each perinatal outcome (figs M and N, appendix 3). These results suggest that the positive linear associations seen when we combined all studies were also present across each of the regions we were able to examine. There is some suggestion that the magnitude of the associations varies by region for some outcomes where these were assessed in several regions. Specifically, the associations with large for gestational age seemed weakest in studies from Asian regions and strongest in studies that were international or from North America, with those from Australasia and Europe in between. But, given the reduced sample sizes within these stratified analyses, it is not possible to determine whether these differences are due to chance.

### Heterogeneity between studies

Figures B-J in appendix 3 show the individual forest plots for each association for fasting, one hour 50 g OGCT post-load, and two hour 75g OGTT. The I^2^ statistic for heterogeneity between the studies for most of the associations was low or 0 (fig 2 and fig A, appendix 3).

## Discussion

In pregnant women without existing diabetes or gestational diabetes mellitus, there are positive linear associations between fasting and post-load glucose concentrations (after 50 g, 75 g, and 100 g loads) and most adverse perinatal outcomes—including caesarean section, induction of labour, large for gestational age, macrosomia, and shoulder dystocia. In general, associations between fasting glucose concentrations and these outcomes were stronger than those of post-load concentrations. Fasting glucose concentration was inversely associated with preterm delivery, but there was no strong evidence of a clear association between post-load concentration and this outcome. In most studies the clinician caring for the woman was likely to have known her glucose concentration and so the findings could have been biased by surveillance/detection bias. When we excluded two studies with four reports in which there was blinding (including the largest and potentially most influential study), however, the results were similar to when we included all studies. When we explored associations by geographical region (Asia, Australasia, Europe, international, and North-America) they showed the same linear pattern of association. Though the 50 g OGCT is not administered after an overnight fast, which invariably introduces a greater degree of variability, we found the same linear associations with this test as with the more controlled 75 g and 100 g OGTT (which are administered after an overnight fast). Thus, our results are robust to different sensitivity analyses based on study quality, population, and type of glucose test. The similarity of results from an OGCT to those from the OGTT suggest that in populations that find fasting difficult, this test could provide some indication of a woman’s response to glucose and the degree of associated risk, though it is important to note that there were relatively few studies for this test and no data available for some of our outcomes.

This detailed systematic review and large scale meta-analysis provides no clear glucose threshold to define gestational diabetes above which risk increases notably across a wide range of clinically relevant pregnancy and perinatal outcomes. The recent IADPSG criteria acknowledged the need to arbitrarily define a threshold for diagnosis. They based this on the point (for fasting, one hour, and two hour post-load 75 g OGTT) at which glucose concentrations above the mean resulted in an odds ratio of at least 1.75 but considered only three outcomes—large for gestational age, large skinfold thickness at birth, and cord blood C peptide. These do not include key clinical outcomes, including the need for induction, caesarean section, neonatal hypoglycaemia, shoulder dystocia, and admission to neonatal intensive care, that obstetricians, midwives, and pregnant women consider important.^51^ Thus, our results show linear associations without thresholds across a range of different populations, with different glucose tests, and for clinically relevant outcomes.

We found no strong evidence of heterogeneity, with low to negligible I^2^ results for all tests. This further supports the robustness of our findings across a wide range of populations, though we acknowledge that our findings would not necessarily generalise to populations in low and middle income countries for which there is little relevant information.

We did not apply the IADPSG odds ratio of 1.75 to define glucose thresholds for gestational diabetes across the wider range of perinatal outcomes explored here for several reasons. Firstly, 1.75 is arbitrary, and we think a range of thresholds ought to be considered. Secondly, application of one odds ratio to all of our outcomes would assume that they are all equally clinically important. But would clinicians and parents consider induction of labour to be as important as shoulder dystocia or an infant requiring neonatal intensive care? The three outcomes that IADPSG used to define thresholds for gestational diabetes (large for gestational age, large thickness skinfold at birth, and cord blood C peptide) were all concerned with the same broad concept of infant adiposity and markers of future risk of obesity in the offspring and so application of the same odds ratio to each of these might be appropriate, but we do not believe it is for the range of outcomes we have examined here. Thirdly, we consider the results from this review should be combined with relevant evidence of treatment effects and economic evaluations, as well as consideration of whether different levels of risk should be applied to different outcomes, to define the optimal clinical and cost-effective thresholds.

### Strengths and limitations

This systematic review and meta-analysis included many studies with varied populations and provides the largest sample of women in whom these associations have been examined. We intentionally had broad inclusion criteria so that we could explore any heterogeneity between study populations and make conclusions relevant to most pregnant women. We found no evidence of heterogeneity overall, but it should be noted that most women came from high income countries. Thus our findings are not necessarily generalisable to lower income settings. Though we wanted to examine the influence of ethnicity on associations, most studies did not provide the detail to allow this. While we found similar patterns of association by geographical region we cannot assume that this reflects ethnicity. For example, the UK Born in Bradford cohort, includes about half white British and half south Asian women.

One of the main limitations of the individual studies was the lack of definite blinding of those who were looking after the pregnant women to the OGTT fasting and post-load glucose concentrations. This could bias the magnitudes of the association towards the null if carers provided advice (about diet for example) or even treatment with oral hypoglycaemics to those women who had borderline high glucose concentrations that did not quite reach the diagnostic criteria for excluding women with gestational diabetes. We tried to explore this in sensitivity analyses of pooled results in those studies that had definitely blinded clinical staff compared with those that had not blinded staff or for which it was unclear whether or not they had blinded them. In general results looked similar in the two groups. Only two studies, however, had definitely blinded staff, and one of these was the largest study—HAPO. The strong associations between fasting glucose concentrations and large for gestational age and macrosomia in the blinded studies compared with other studies could reflect blinding, but it could also be a chance finding considering the number of comparisons undertaken in this sensitivity analysis. Given that this analysis is comparing just one or two studies with all others it could also be driven by other differences. Importantly, the difference is small and does not alter our overall conclusion regarding the linear dose-response nature of the associations between glucose concentrations and a wide range of clinically important perinatal outcomes.

The inclusion of women with a diagnosis of gestational diabetes would have affected the estimates of the association between glucose concentration and outcomes as these women would be treated to reduce their concentration; they were therefore excluded. Although we found no evidence of a curvilinear association between glucose and outcomes at concentrations below current treatment thresholds, the possibility exists that risks might increase substantially at concentrations exceeding them.

The increased identification of women resulting from lowering glucose thresholds to diagnose gestational diabetes has resource implications for maternity services in terms of antenatal care (OGTTs, treatments, induction of labour), intrapartum care (caesarean section), and short and longer term postnatal care (infant care, screening for type 2 diabetes). Costs are likely to be greater for identification and treatment strategies that use lower glucose thresholds if care packages are unchanged. Because there is a graded linear association between maternal glucose concentration and risk of perinatal outcomes, risk of these outcomes could be reduced if thresholds are lowered. There are no trials using these new thresholds, however, and no robust evidence that the longer term risk of obesity would be improved.^52^

### Recommendations for research

Considering all eligible evidence, it is clear that the association between glucose concentration and a wide range of clinically relevant adverse perinatal outcomes is linear and that there is no threshold above which odds substantially increase in high income countries. With the exception of large well conducted studies in low and middle income countries, we recommend that further studies of the nature of the association of gestational glucose with perinatal outcomes are not required. We believe that studies in low and middle income countries are important, and this might be particularly the case for sub-Saharan Africa, where there seems to have been no studies to date but where the prevalence of diabetes is increasing and possibly has a different phenotype to that seen in Western high income countries and where perinatal outcomes also have different presentations.^53^ ^54^ Also there are few studies in South Asia, but again diabetes is an increasing problem here and could influence perinatal outcomes in a different way to that seen in European origin populations, as suggested by our earlier results in Born in Bradford.^13^

As noted above, rather than apply an arbitrary level of risk, such as an odds ratio of 1.75, to all of the clinically relevant outcomes we have examined here, we believe that future research needs to combine our results with robust evidence from well conducted randomised trials (and meta-analyses of those) of treatment effects on adverse outcomes related to gestational diabetes. Economic evaluations and research are required to determine what relative importance women, their partners, and care givers attribute to the different outcomes to determine the level at which clinical and cost effectiveness is maximised.

**What is already known on this topic**

Gestational diabetes (GDM) is associated with increased risk of a range of adverse perinatal outcomes and can affect the longer term health of mother and offspring
Treatment seems to reduce the risk of these outcomes, but the optimal glucose threshold to define gestational diabetes is unknown
Published thresholds for fasting and post-load glucose thresholds for diagnosing gestational diabetes are based on results from one multicentre study that considered large for gestational age, large skinfold thickness at birth, and cord blood C peptide)
Not all outcomes that pregnant women and clinicians would consider to be clinically important (including labour induction, caesarean section, neonatal hypoglycaemia, shoulder dystocia, and the need for neonatal intensive care) have been taken into account

**What this study adds**

There is a consistent graded linear association between glucose concentrations and clinically relevant perinatal outcomes (caesarean section, induction of labour, large for gestational age, macrosomia, and shoulder dystocia), with no clear threshold
These patterns were robust to sensitivity analyses exploring the impact of study quality and type of glucose exposure and across geographical regions (studies from Asia, Australasia, Europe, North America and international (multicentre) studies)
There is currently no evidence from sub-Saharan Africa regarding the association between of gestational glucose and perinatal outcomes and little evidence from other low and middle income countries

## Web Extra

Extra material supplied by the author

Appendix 1: Search strategy in Medline

Appendix 2: Details of statistical methods and models

Appendix 3: Supplementary tables, forest plots, and references

Appendix 4: Risks of all outcomes against glucose concentration for each study, test, and timing

## Notes

We thank Julie Glanville and Mick Arber of the York Health Economics Consortium who carried out the searches.

Ethical approval: The Born in Bradford study was approved by the Bradford research ethics committee (07/H1302/112). All participants provided informed written consent.

Data sharing: Extracted data are available on request to the corresponding author.

Transparency: The lead author affirms that the manuscript is an honest, accurate, and transparent account of the study being reported; that no important aspects of the study have been omitted; and that any discrepancies from the study as planned (and, if relevant, registered) have been explained.
