# Supplementary material for: Hyperglycaemia and risk of adverse perinatal outcomes: systematic review and meta-analysis
Source: BMJ. 2016 Sep 13;354:i4694. doi: 10.1136/bmj.i4694 (PMC5021824; doi:10.1136/bmj.i4694)
Supplement: Supplementary file 1 — Appendix 1: Search strategy in Medline [file fard032237.ww1_default.pdf]

Appendix 1: Full MEDLINE Search strategy [posted as supplied by author]

1 (pregnancy adj4 diabetes).ti,ab. (4082)
2 (gestational adj4 diabetes).ti,ab. (8108)
3 exp DIABETES, GESTATIONAL/ (7439)
4 gdm.ti,ab. (3272)
5 (glucose adj4 (pregnan* or gestation* or natal or maternal)).ti,ab. (3469)
6 1 or 2 or 3 or 4 or 5 (15075)
7 macrosomia.ti,ab. (2314)
8 exp FETAL MACROSOMIA/ (1826)
9 7 or 8 (3157)
10 exp BIRTH INJURIES/ (4937)
11 ((perinatal or labor or labour or birth) adj4 trauma).ti,ab. (1355)
12 ((perinatal or labor or labour or birth) adj4 injur*).ti,ab. (2542)
13 ((perinatal or labor or labour or birth) adj4 complication*1).ti,ab. (4372)
14 exp OBSTETRIC LABOR COMPLICATIONS/ (53369)
15 *DYSTOCIA/ (1902)
16 (shoulder adj4 dystocia).ti,ab. (1021)
17 (fracture*1 adj4 clavicle*1).ti,ab. (1218)
18 (fracture*1 adj4 humerus).ti,ab. (3451)
19 (fracture*1 adj4 shoulder*1).ti,ab. (753)
20 (fracture*1 adj4 arm*1).ti,ab. (454)
21 "erb* palsy".ti,ab. (185)
22 neuropath*.ti,ab. (97784)
23 exp BRACHIAL PLEXUS NEUROPATHIES/ (2817)
24 10 or 11 or 12 or 13 or 14 or 15 or 16 or 17 or 18 or 19 or 20 or 21 or 22 or 23 (168258)
25 (preeclampsia or pre-eclampsia).ti,ab. (20669)
26 exp PRE-ECLAMPSIA/ (24509)
27 25 or 26 (31679)
28 (heart adj4 (disorder*1 or disease*1)).ti,ab. (142562)
29 (cardiovascular adj4 (disorder*1 or disease*1)).ti,ab. (119950)
30 (cardiac adj4 (disorder*1 or disease*1)).ti,ab. (26958)
31 exp CARDIOVASCULAR DISEASES/ (1944605)
32 exp HEART DISEASES/ (922916)
33 28 or 29 or 30 or 31 or 32 (2024083)
34 exp HYPOGLYCEMIA/ (22500)
35 hypoglyc*.ti,ab. (42033)
36 34 or 35 (48692)
37 exp DIABETES MELLITUS, TYPE 2/ (90640)
38 (("type 2" or "' type AND two" or "type II") adj4 diabet*).ti,ab. (87156)
39 37 or 38 (121847)
40 exp OBESITY/ (152662)
41 (obesity or obese or bmi or "body mass" or overweight).ti,ab. (311123)

42 40 or 41 (343012)

43 9 or 24 or 27 or 33 or 36 or 39 or 42 (2561831)

44 (offspring or son*1 or daughter*1 or child or children or pediatric*1 or paediatric*1).ti,ab. (1177569)

45 exp CHILD OF IMPAIRED PARENTS/ (4392)

46 exp CHILD/ (1595153)

47 (maternal or mother*2).ti,ab. (288181)

48 exp MOTHERS/ (27857)

49 44 or 45 or 46 or 47 or 48 (2246955)

50 43 and 49 (274768)

51 6 and 50 (4840)

52 51 not (animals/ not humans/) (4622)
