# Supplementary material for: Hyperglycaemia and risk of adverse perinatal outcomes: systematic review and meta-analysis
Source: BMJ. 2016 Sep 13;354:i4694. doi: 10.1136/bmj.i4694 (PMC5021824; doi:10.1136/bmj.i4694)
Supplement: Supplementary file 2 — Appendix 2: Details of statistical methods and models [file fard032237.ww2_default.pdf]

Appendix 2: Statistical methods [posted as supplied by author]

To model the associations between outcomes and glucose levels a log-linear relationship between risk and outcome was assumed; that is, the log odds of the outcome was assumed to vary linearly with glucose level. This association was modelled separately for each outcome and glucose test. Formally the models had the form:

$$\log\left(\frac{p_{ijkl}}{1-p_{ijkl}}\right) = \phi_{ijkl} + \theta_{ijkl}G_{ijkl}$$
$$\theta_{ijkl} \sim N\left(\theta^{*}_{jkl}, \tau^{2}_{jkl}\right)$$

where i indicates study, j glucose test (eg. fasting 75g OGTT), k the outcome of interest (eg. macrosomia) and l the glucose category. Then p_{ijkl} is the probability of having the outcome in the relevant glucose category, G_{ijkl} is the typical glucose level in that category. Hence ϕ_{ijkl} is the baseline log odds of the outcome in study I, which are assumed to be independent across stuidies. Also, $\theta_{i\ ijkl}$ is the association between glucose and outcome, in terms of the log odds of outcome per 1 mmol/L increase in glucose, assumed to have a random effect across studies to allow for heterogeneity in the trend. The model was fitted in R using the lme4 package for mixed effect regression modelling.

For outcomes reported in only one study the same logistic regression model was used without the meta-analysis across studies or the random effects. That is:

$$\log\left(\frac{p_{l}}{1-p_{l}}\right) = \phi_{l} + \theta_{l}G_{l}$$

To test the assumption of linearity a term in glucose squared was added to each model:

$$\log\left(\frac{p_{ijkl}}{1-p_{ijkl}}\right) = \phi_{ijkl} + \theta_{ijkl}G_{ijkl} + \gamma_{jkl}G^{2}_{ijkl}$$
$$\theta_{ijkl} \sim N\left(\theta^{*}_{jkl}, \tau^{2}_{jkl}\right)$$

With the glucose squared terms γ modelled as fixed effects. Any deviation from linearity would be indicated by finding a statistically significant γ term.
