# Supplementary material for: Hyperglycaemia and risk of adverse perinatal outcomes: systematic review and meta-analysis
Source: BMJ. 2016 Sep 13;354:i4694. doi: 10.1136/bmj.i4694 (PMC5021824; doi:10.1136/bmj.i4694)
Supplement: Supplementary file 3 — Appendix 3: Supplementary tables, forest plots, and references [file fard032237.ww3_default.pdf]

Appendix 3: Supplementary tables and forest plots [posted as supplied by author]

Table A. Results of the risk of bias assessment

Study	Year of publication	Prospective or retrospective	Representative population	Loss to follow up	Consistent glucose measurement	Consistent outcome measurement	'Blinding'		Selective reporting	Adjusted results presented
							of glucose measurements	of outcomes		
Atlantic DIP	N/A	P	Low	Low	Low	Low	High	High	Low	Low
Aris	2014	P	Low	Low	Low	Low	Unclear	Unclear	Low	Low
BiB	N/A	P	Low	Low	Low	Low	High	High	Low	Low
Carr	2011	R	Low/moderate	Low	Low	Low	High	High	Low	Low
Chadna	2006	R	Unclear	Low	Unclear	Unclear	High	High	Unclear	High
Cheng	2007	R	Low	Low	Low	Unclear	High	High	Unclear	Low
Figueroa	2013	Secondary analysis of RCT data	Low (but subset of trial)	Low	Low	Low	Unclear	Unclear	Low	Low
HAPO	2008	P	Low	Low	Low	Low	Low	Low	Low	Low
HAPO	2010	P	Low	Low	Low	Low	Low	Low	Low	Low
Hillier	2008	Unclear	Low	Low	Low	Unclear	Unclear	Unclear	Unclear	Low
Jensen	2001	R	High (higher risk group)	Low	Low	Low	High	High	Low	High
Kerenyi	2009	Unclear	Low	Low	Low	Low	Unclear	Unclear	Unclear	Low
Landon	2011	Secondary analyses of RCT data	Low (subset of trial)	Low	Low	Low	Unclear	Low	Low	Low
Lao	2003	R	Low (Chinese)	Low	Low	Low	High	High	Low	High
Little	1990	P	Low	Low	Low	Unclear	Unclear	Unclear	Unclear	High
Lurie	1998	P	Low	Low	Low	Low	Unclear	Low	Low	High
Metzger	2010	P	Low	Low	Low	Low	Low	Low	Low	Low
Moses	1995	P	Low	Unclear	Low	Low	Unclear	Unclear	Low	High
Ong	2008	R	Low	Low	Low	Unclear	High	High	Unclear	High
Pettitt	1980	P	High (Pima Indian)	Low	Low	Low	Unclear	Unclear	Unclear	High
Riskin-Mashia	2009	R	Low	Low	Low	Low	High	High	Low	Limited adjustment
Savona-Ventura	2010	R	Low	Low	Low	Unclear	High	High	Unclear	High
Scholl	2001	P	Low	Low	Low	Low	Unclear	Unclear	Low	Low
Sermer	1995	P	Low	Low	Low	Low	Low	Low	Low	High
Tallarigo	1986	Unclear	Low	Low	Low	Low	Unclear	Unclear	Low	High
Witter	1988	R	Low, but young age group	Low	Low	Low	High	High	Low	High
Yee	2011	R	Low	Low	Low	Low	High	High	Low	Low

R=retrospective, P=prospective,

Table B. Analysis testing for linearity of association between glucose levels and outcomes

Outcome	Number of studies	Log odds ratio of glucose squared	95% CI	P-value
**Fasting 75g OGTT**				
C-section	6	-0.115	(-0.25 - 0.02)	0.1
Induction	3	-0.197	(-0.52 - 0.13)	0.23
Instrumental birth	3	0.107	(-0.21 - 0.42)	0.5
LGA	7	-0.02	(-0.16 - 0.12)	0.77
Macrosomia	6	-0.18	(-0.39 - 0.03)	0.09
Neonatal hypoglycemia	2	0.29	(0.05 - 0.53)	0.02
PIH/Pre-eclampsia	3	0.461	(0.03 - 0.9)	0.04
Pre-eclampsia	4	-0.005	(-0.28 - 0.27)	0.97
Preterm birth	3	0.577	(0.09 - 1.07)	0.02
Shoulder dystocia	4	-0.142	(-1.06 - 0.78)	0.76
**50g OGCT**				
C-section	7	-0.029	(-0.07 - 0.01)	0.18
Instrumental birth	2	-0.008	(-0.08 - 0.07)	0.84
LGA	4	-0.044	(-0.1 - 0.01)	0.11
Macrosomia	7	-0.004	(-0.02 - 0.02)	0.69
Neonatal hypoglycemia	3	0.047	(-0.18 - 0.27)	0.68
Pre-eclampsia	6	-0.082	(-0.15 - -0.02)	0.01
Preterm birth	2	0.021	(-0.05 - 0.09)	0.55
Shoulder dystocia	2	-0.113	(-0.25 - 0.03)	0.12
**2 hour 75g OGTT**				
C-section	9	-0.016	(-0.03 – 0.00)	0.06
Induction	3	0.006	(-0.04 - 0.05)	0.81
Instrumental birth	4	-0.01	(-0.05 - 0.03)	0.65
LGA	11	0.004	(-0.01 - 0.02)	0.67
Macrosomia	7	0.006	(-0.03 - 0.05)	0.77
Neonatal hypoglycemia	3	0.002	(-0.02 - 0.03)	0.91
PIH/Pre-eclampsia	3	0.02	(-0.07 - 0.11)	0.67
Pre-eclampsia	4	-0.026	(-0.05 – 0.00)	0.05
Preterm birth	6	0.009	(-0.05 - 0.07)	0.78
Shoulder dystocia	5	-0.067	(-0.19 - 0.06)	0.29

Figure A. Odd ratios for outcomes at one and two-hour and combined for the 75g and 100g post-load OGTT

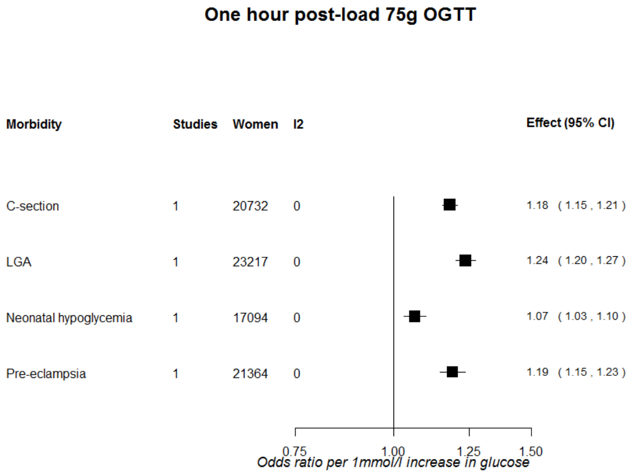

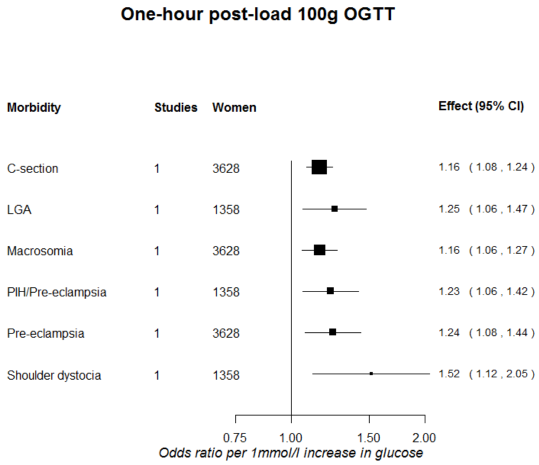

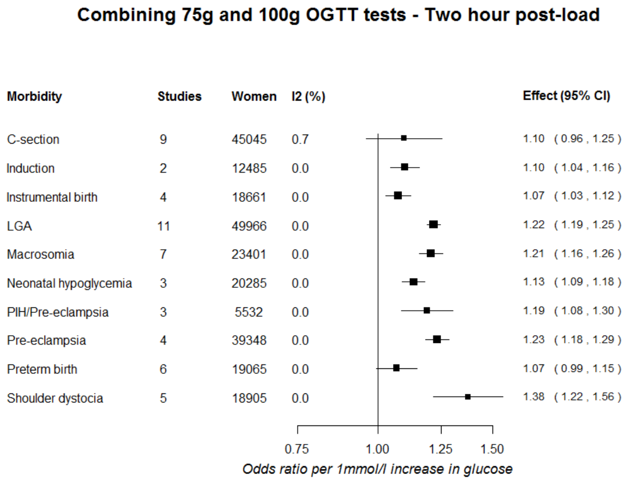

Figure B. Associations for fasting, one-hour post-load 50g OGCT and two-hour post-load 75g OGTT and Caesarean section

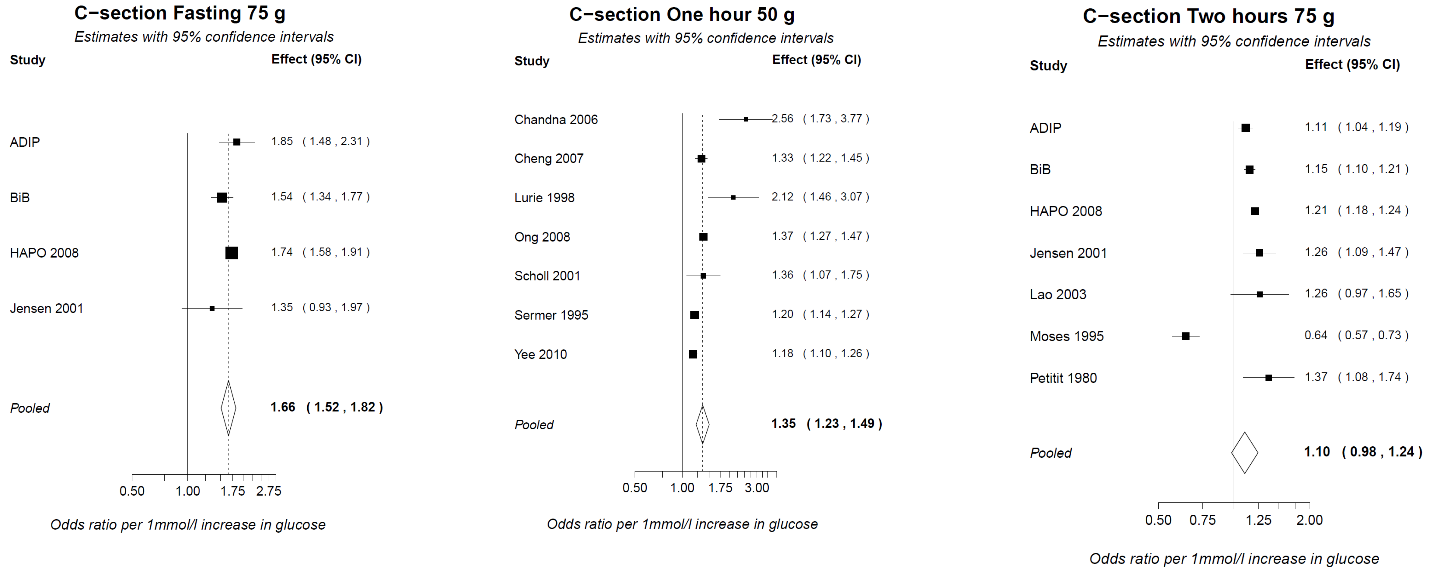

Figure C. Associations for fasting, one-hour post-load 50g OGCT and two-hour post-load 75g OGTT and Large for gestational age

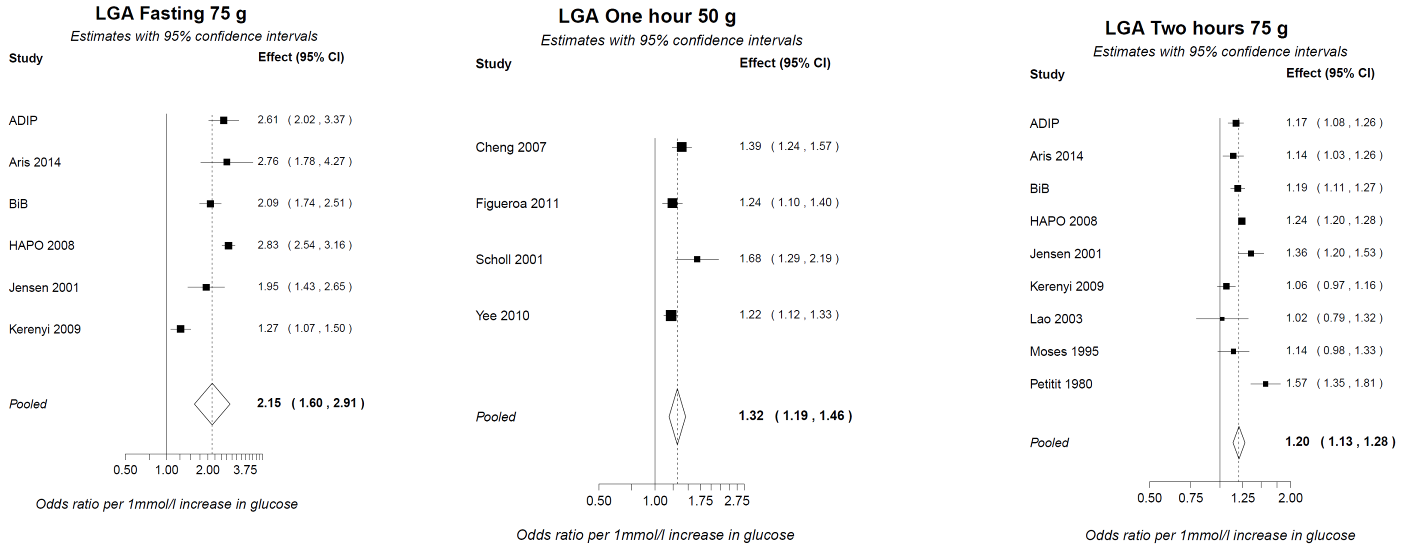

Figure D. Associations for fasting, one-hour post-load 50g OGCT and two-hour post-load 75g OGTT and macrosomia

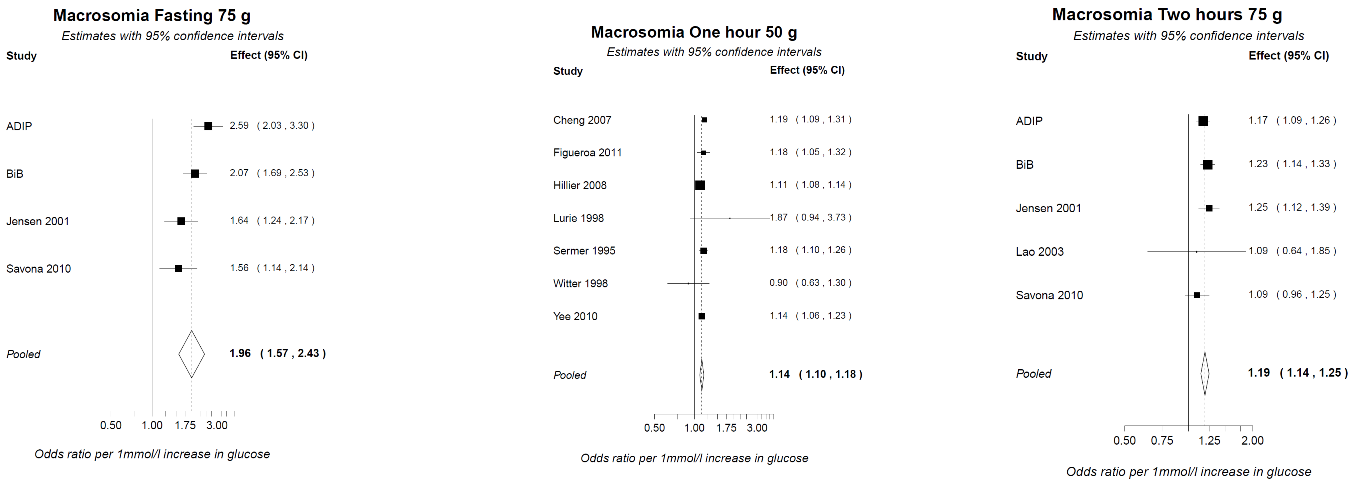

Figure E. Associations for fasting, one-hour post-load 50g OGCT and two-hour post-load 75g OGTT and induction of labour

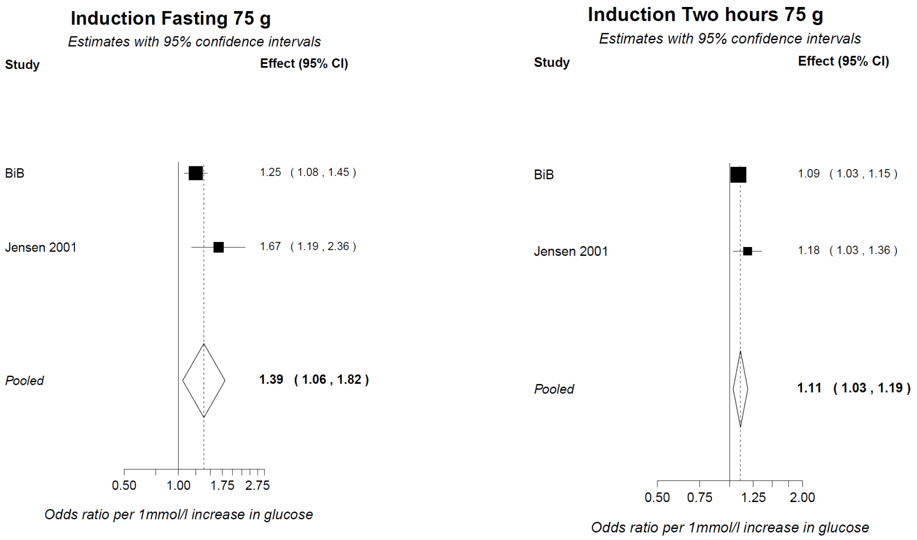

Figure F. Associations for fasting, one-hour post-load 50g OGCT and two-hour post-load 75g OGTT and instrumental birth

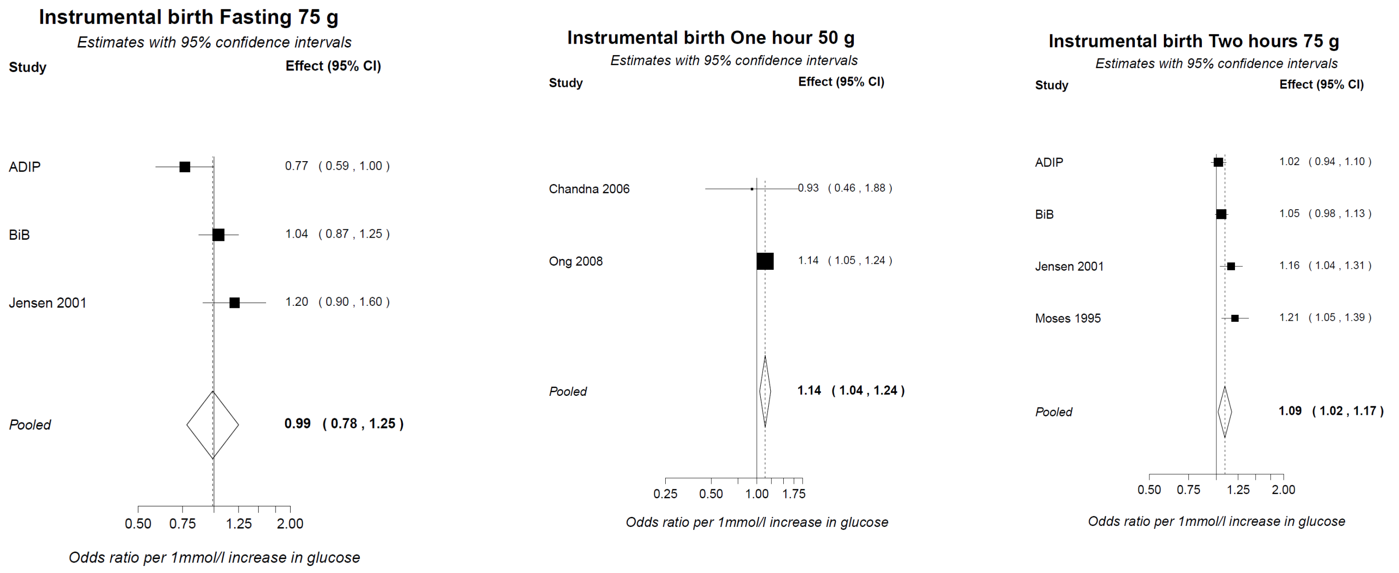

Figure G. Associations for fasting, one-hour post-load 50g OGCT and two-hour post-load 75g OGTT and macrosomia

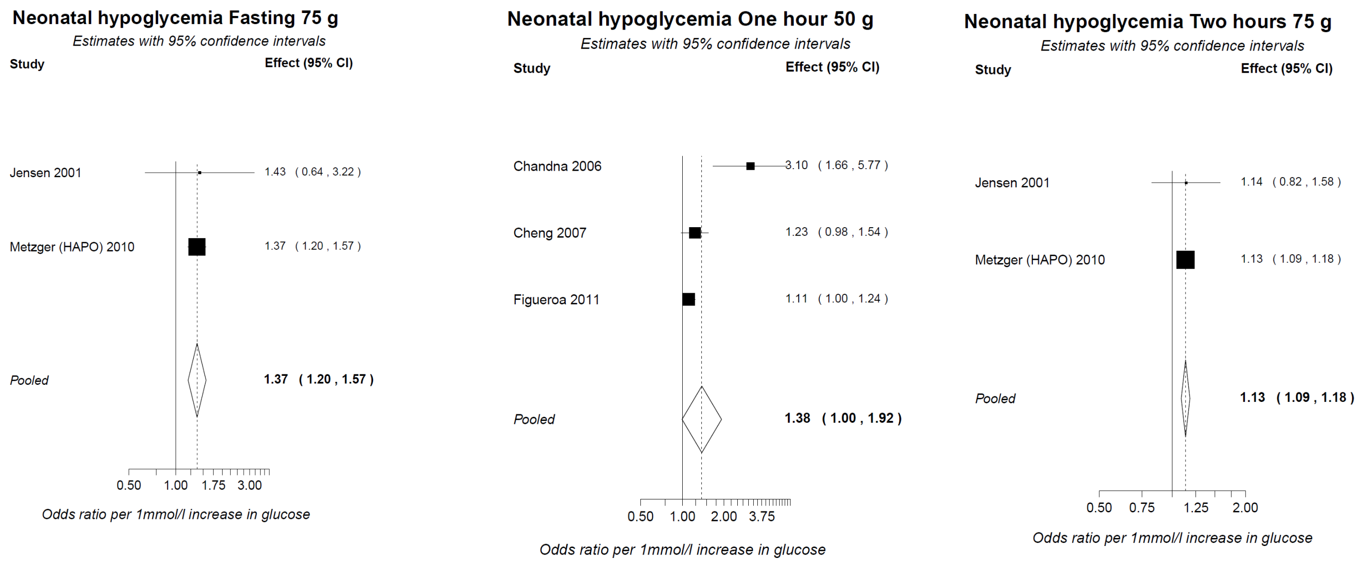

Figure H. Associations for fasting, one-hour post-load 50g OGCT and two-hour post-load 75g OGTT and pre-eclampsia

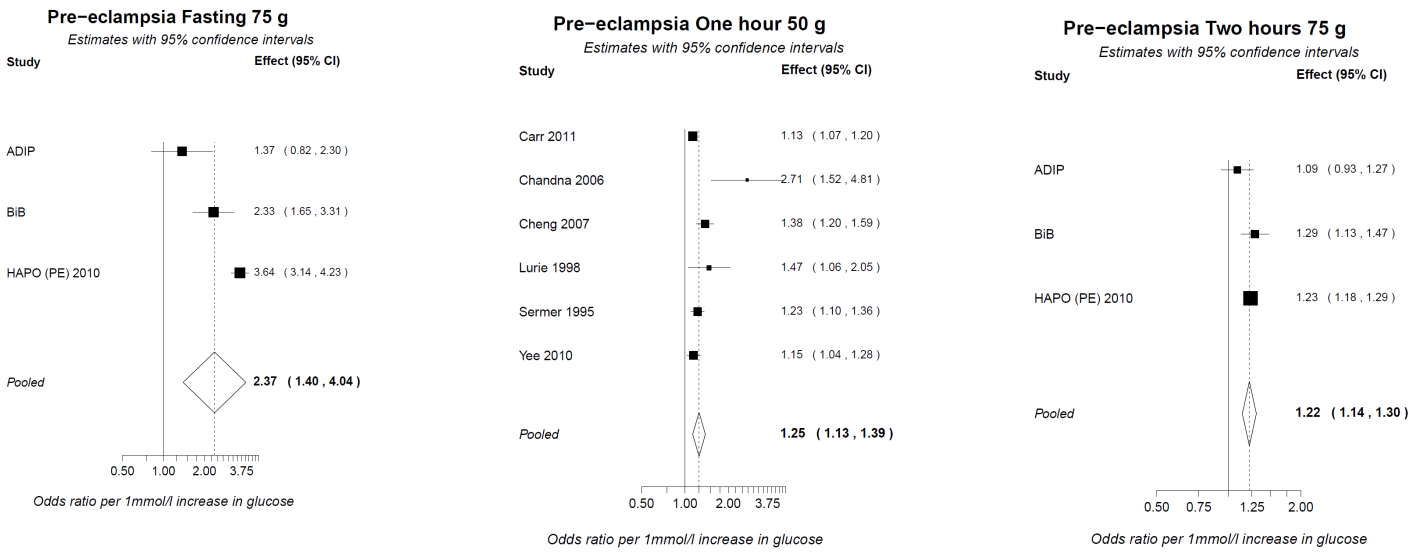

Figure I. Associations for fasting, one-hour post-load 50g OGCT and two-hour post-load 75g OGTT and preterm birth

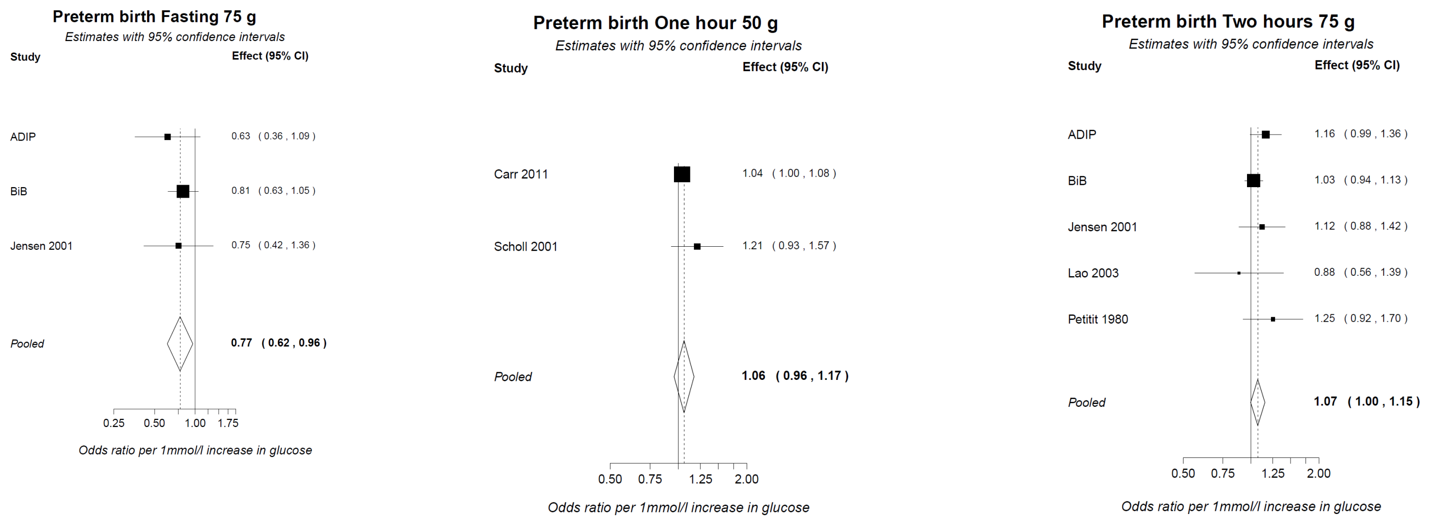

Figure J. Associations for fasting, one-hour post-load 50g OGCT and two-hour post-load 75g OGTT and shoulder dystocia

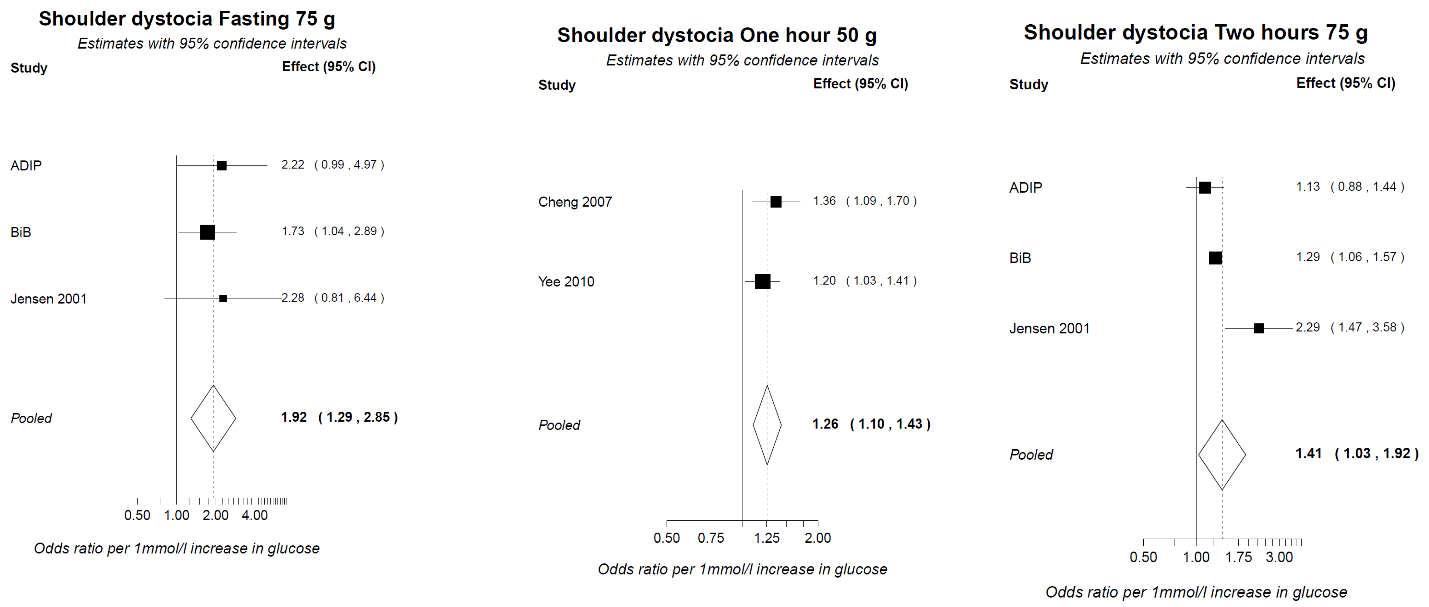

Figure K. Odd ratios for outcomes at fasting 75g and 100g OGTT combined and grouped by whether blinded or unblinded/unclear

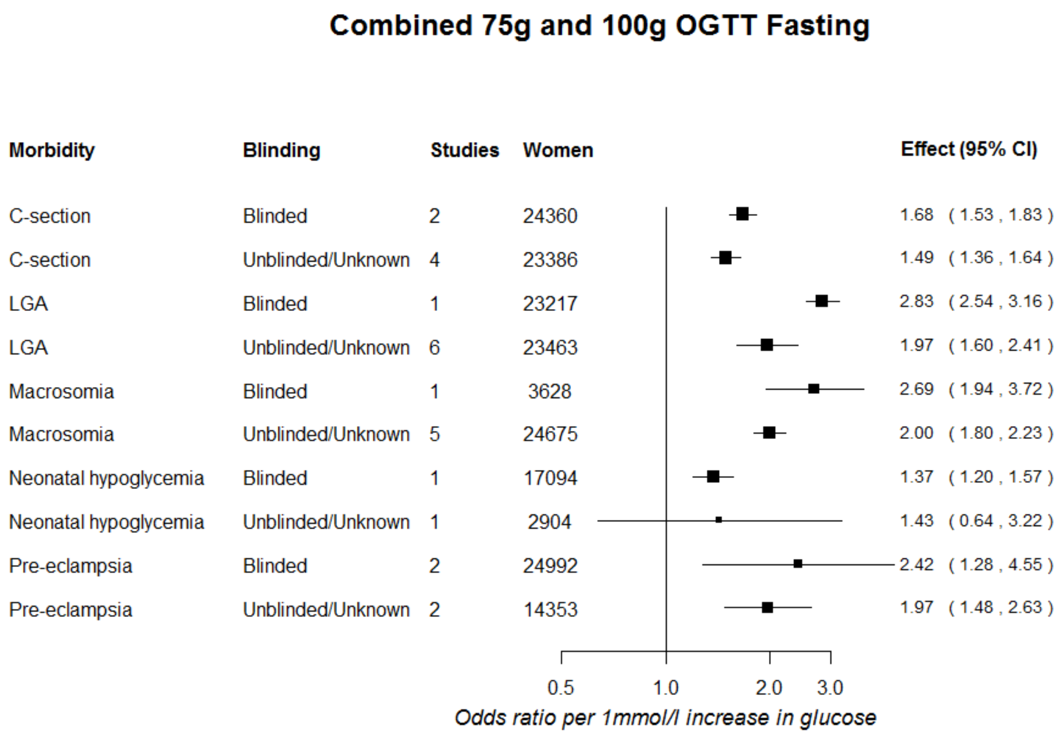

Figure L. Odd ratios for outcomes at two-hour post-load 75g and 100g OGTT combined and grouped by whether blinded or unblinded/unclear

Combined 75g and 100g OGTT Two hours

Morbidity	Blinding	Studies	Women	Effect (95% CI)
C-section	Blinded	2	24360	1.21 (1.17 , 1.24)
C-section	Unblinded/Unknown	7	20685	1.07 (0.90 , 1.27)
LGA	Blinded	1	23217	1.24 (1.20 , 1.28)
LGA	Unblinded/Unknown	10	26749	1.19 (1.15 , 1.24)
Macrosomia	Blinded	1	3628	1.26 (1.12 , 1.41)
Macrosomia	Unblinded/Unknown	6	19773	1.20 (1.15 , 1.25)
Neonatal hypoglycemia	Blinded	1	17094	1.13 (1.09 , 1.18)
Neonatal hypoglycemia	Unblinded/Unknown	2	3191	1.13 (0.86 , 1.48)
Pre-eclampsia	Blinded	2	24992	1.24 (1.19 , 1.30)
Pre-eclampsia	Unblinded/Unknown	2	14356	1.20 (1.08 , 1.33)

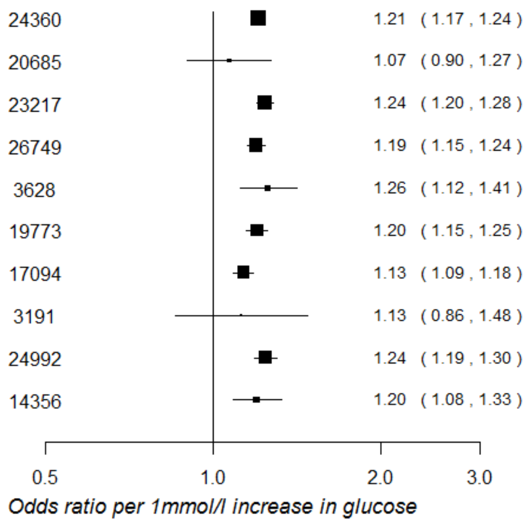

Figure M. Odd ratios for outcomes at fasting 75g and 100g OGTT combined and grouped by region

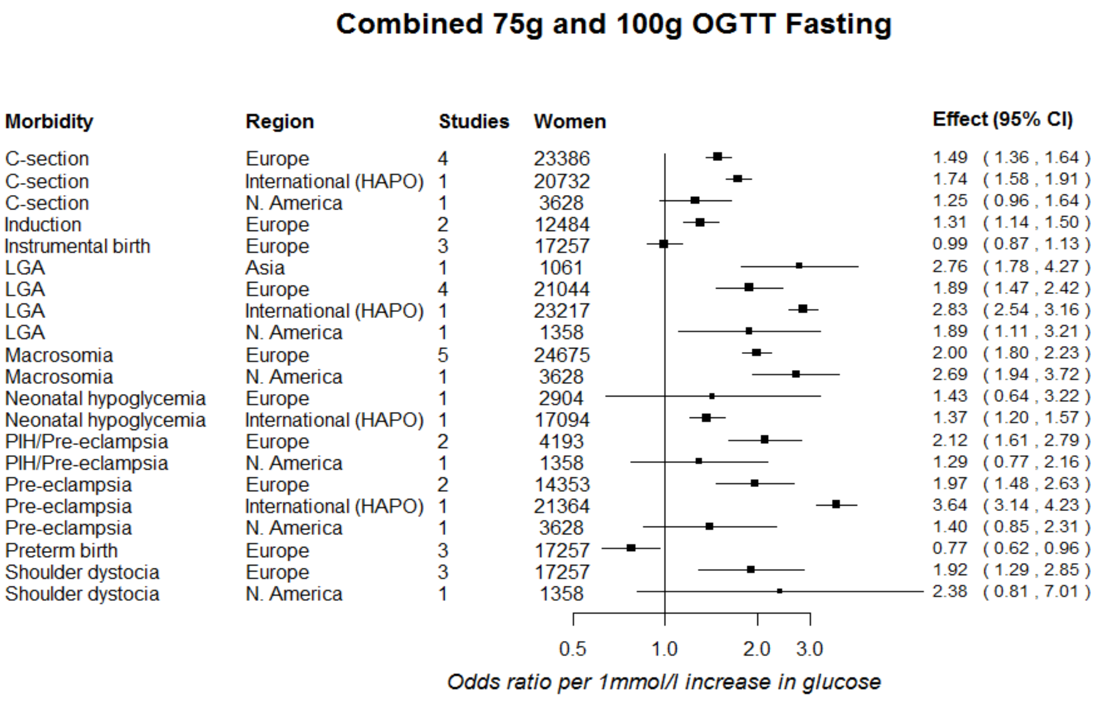

N. America (North America= Canada and USA),[1] Multinational,[2-4] Asia (Singapore, China),[5,6] Europe (Denmark, Hungry, Malta UK),[7-11] Australia[12]

Figure N. Odd ratios for outcomes at two-hour post-load 75g OGTT grouped by region

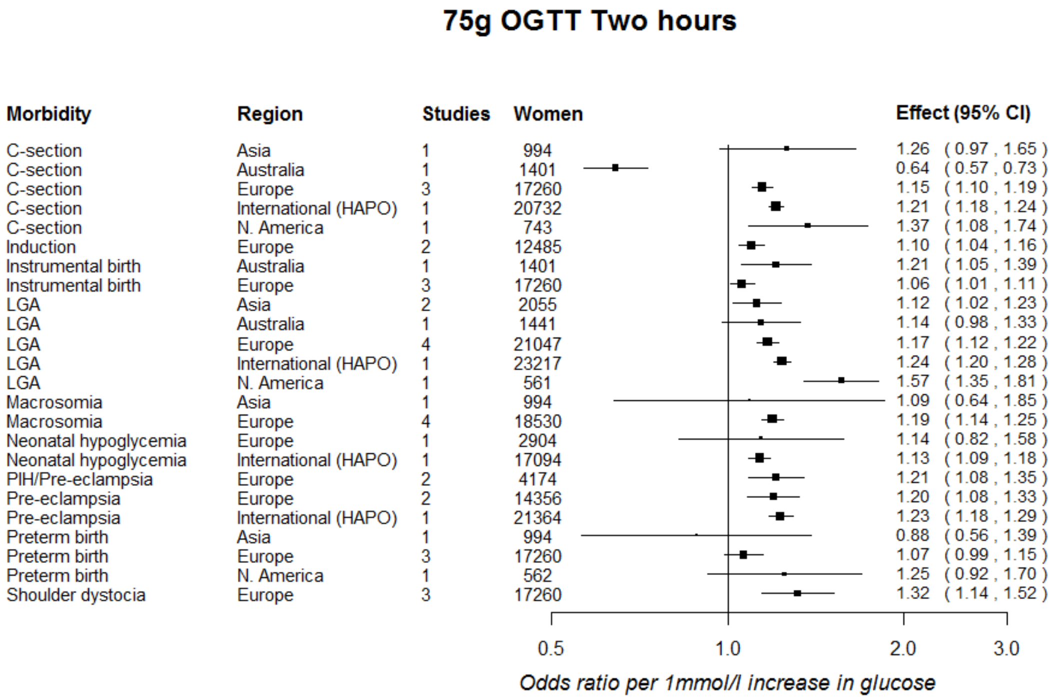

N. America (North America= Canada and USA),[1] Multinational,[2-4] Asia (Singapore, China),[5,6] Europe (Denmark, Hungry, Malta UK),[7-11] Australia[12]

References

1. Pettitt DJ, Knowler WC, Baird HR, Bennett PH. Gestational diabetes: infant and maternal complications of pregnancy in relation to third-trimester glucose tolerance in the Pima Indians. *Diabetes Care*. 1980; 3(3): 458-64.
2. HAPO Hyperglycemia and Adverse Pregnancy Outcomes. *N Engl J Med*. 2008; 358: 1991-2002.
3. HAPO Study Cooperative Research Group. Hyperglycemia and adverse pregnancy outcome (HAPO) study: preeclampsia. *Am J Obstet Gynecol*. 2010; 202(3): e1-e7.
4. Metzger BE, Persson B, Lowe LP, Dyer AR, Cruickshank JK, Deerochanawong C, et al. Hyperglycemia and Adverse Pregnancy Outcome Study: Neonatal Glycemia. *Pediatrics*. 2010; 126(6): e1545-e52.
5. Aris IM, Soh SE, Tint MT, Liang S, Chinnadurai A, Saw SM, et al. Effect of Maternal Glycemia on Neonatal Adiposity in a Multiethnic Asian Birth Cohort. *J Clin Endocrinol Metab*. 2014; 99(1): 240–7.
6. Lao TT, Ho LF. Does maternal glucose intolerance affect the length of gestation in singleton pregnancies? *J Soc Gynecol Invest*. 2003; 10(6): 366-71.
7. Jensen DM, Damm P, Sorensen B, Molsted-Pedersen L, Westergaard JG, Klebe J, et al. Clinical impact of mild carbohydrate intolerance in pregnancy: a study of 2904 nondiabetic Danish women with risk factors for gestational diabetes mellitus. *Am J Obstet Gynecol*. 2001; 185(2): 413-9.
8. Kerényi Z, Tamás G, Kivimäki M, Péterfalvi A, Madarász E, Bosnyák Z, et al. Maternal Glycemia and Risk of Large-for-Gestational-Age Babies in a Population-Based Screening. *Diabetes Care*. 2009; 32(12): 2200-5.
9. Savona-Ventura C, Craus J, Vella K, Grima S. Lowest threshold values for the 75g oral glucose tolerance test in pregnancy. *Malta Med J*. 2010; 22(1): 18-20.
10. Wright J. Born in Bradford cohort. *Personal communication*. 2014.
11. Dunne F. Atlantic Diabetes in Pregnancy cohort. *Personal communication*. 2014.
12. Moses RGF, Calvert DP. Pregnancy Outcomes in Women Without Gestational Diabetes Mellitus Related to the Maternal Glucose Level: Is there a continuum of risk? *Diabetes Care*. 1995; 18(12): 1527-33.
