# Supplementary material for: Hyperglycaemia and risk of adverse perinatal outcomes: systematic review and meta-analysis
Source: BMJ. 2016 Sep 13;354:i4694. doi: 10.1136/bmj.i4694 (PMC5021824; doi:10.1136/bmj.i4694)

Appendix 4: Risks of all outcomes against glucose concentrations for each study, glucose test, and timing [posted as supplied by author]

Fig A Risk of caesarean section against glucose concentration for each study, glucose test, and timing

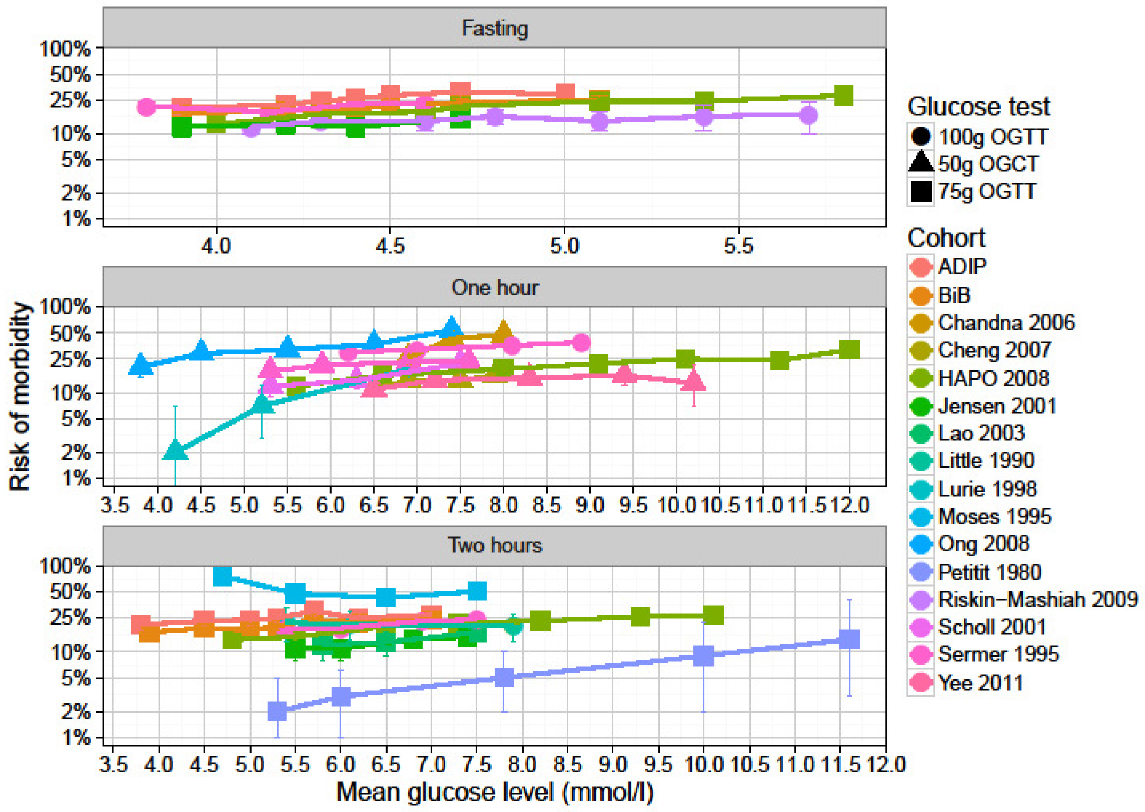

Fig B Risk of macrosomia against glucose concentration for each study, glucose test, and timing

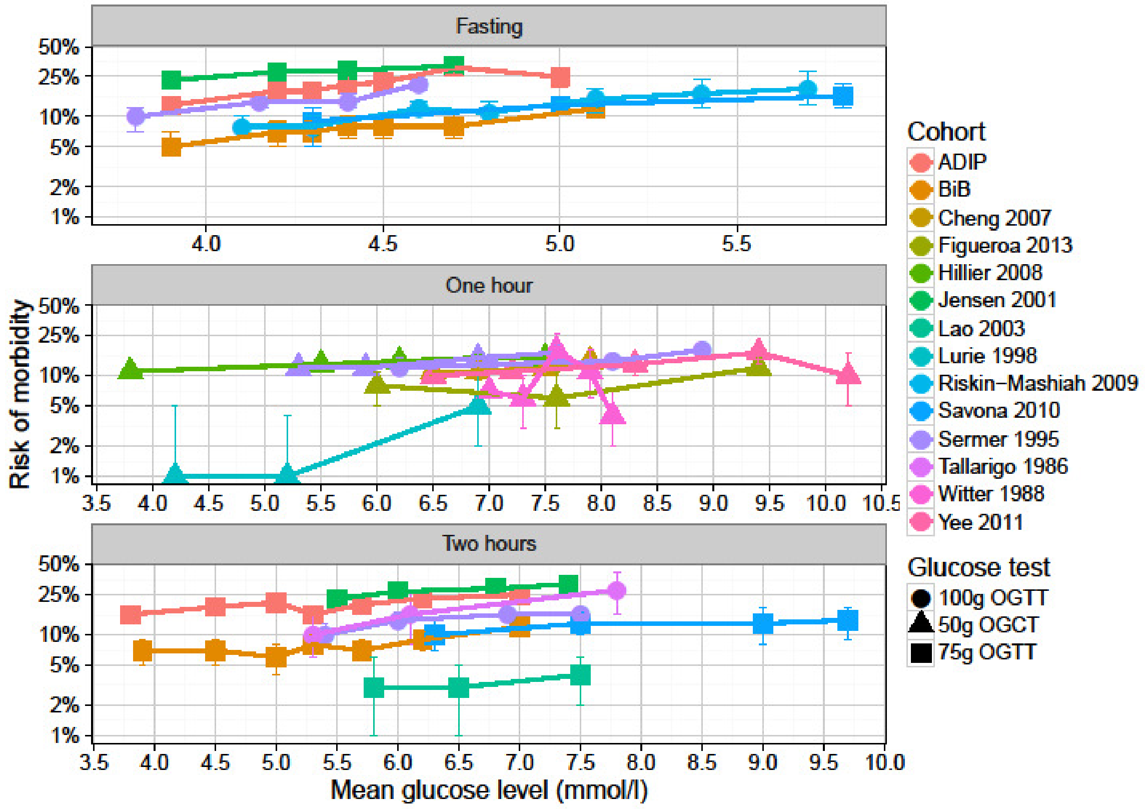

Fig C Risk of large for gestational age against glucose concentration for each study, glucose test, and timing

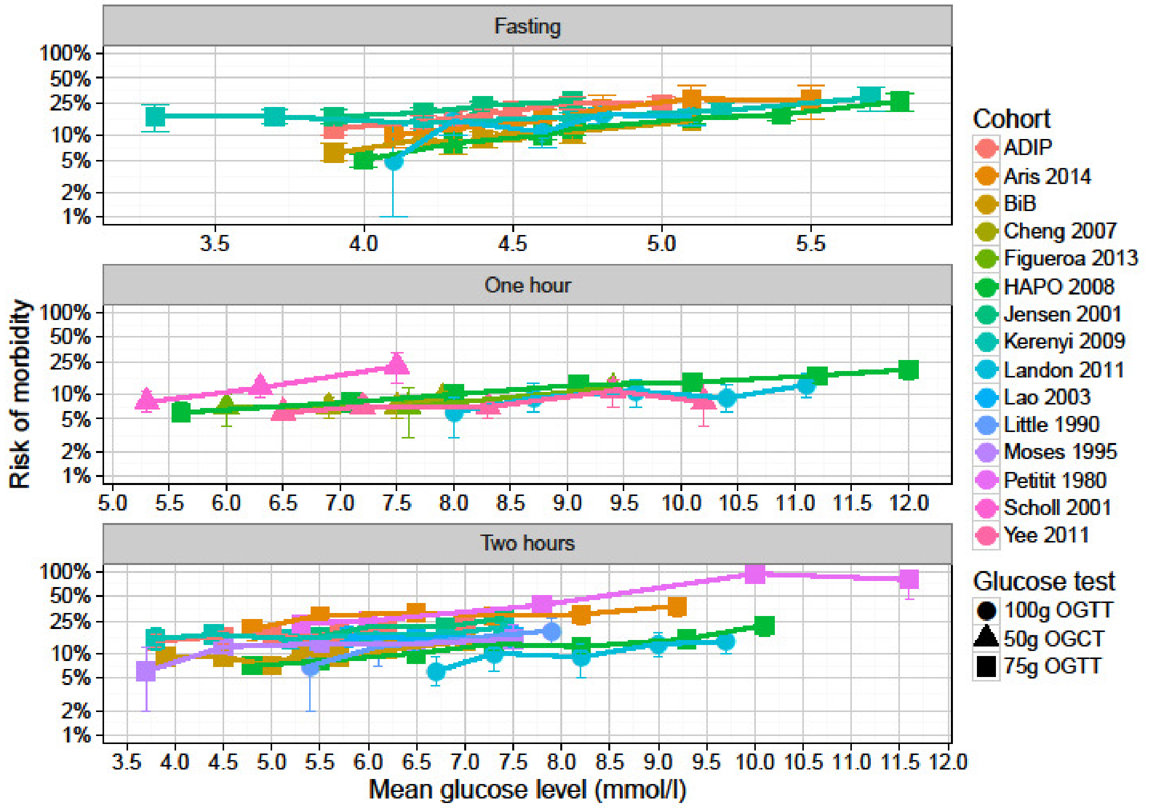

Fig D Risk of pre-eclampsia against glucose concentration for each study, glucose test, and timing

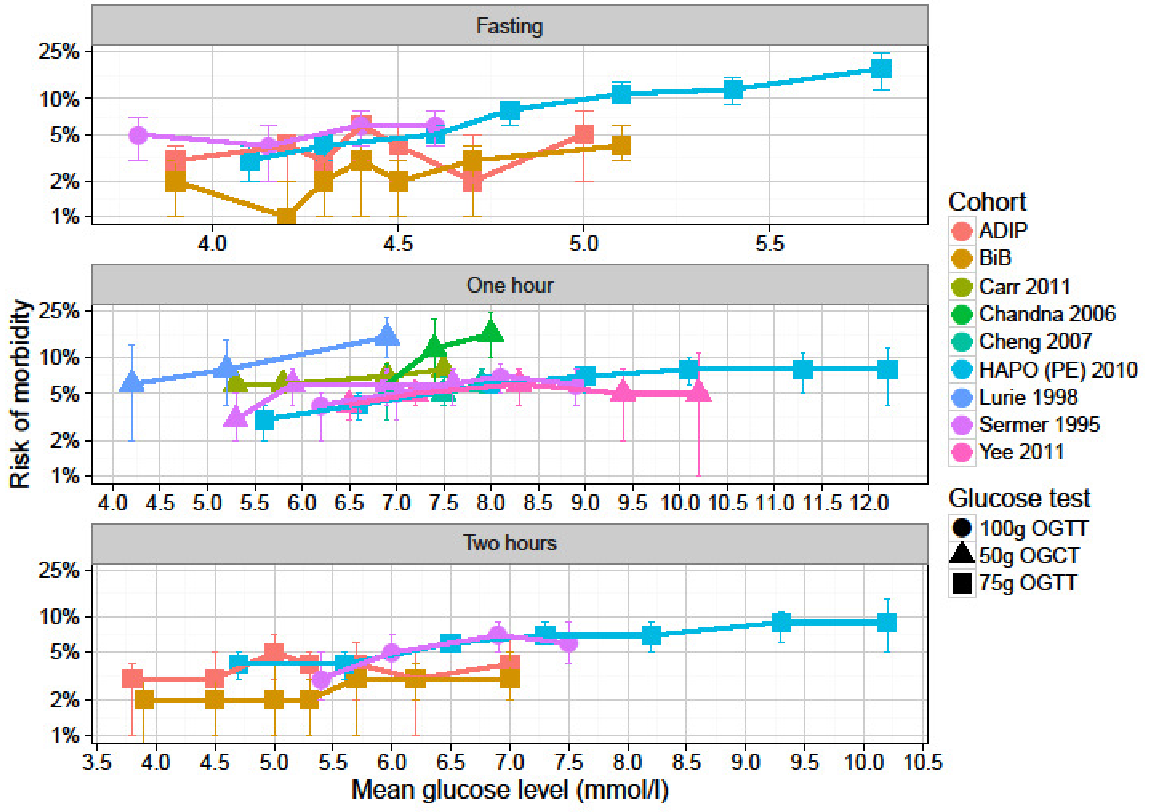

Fig E Risk of instrumental birth against glucose concentration for each study, glucose test, and timing

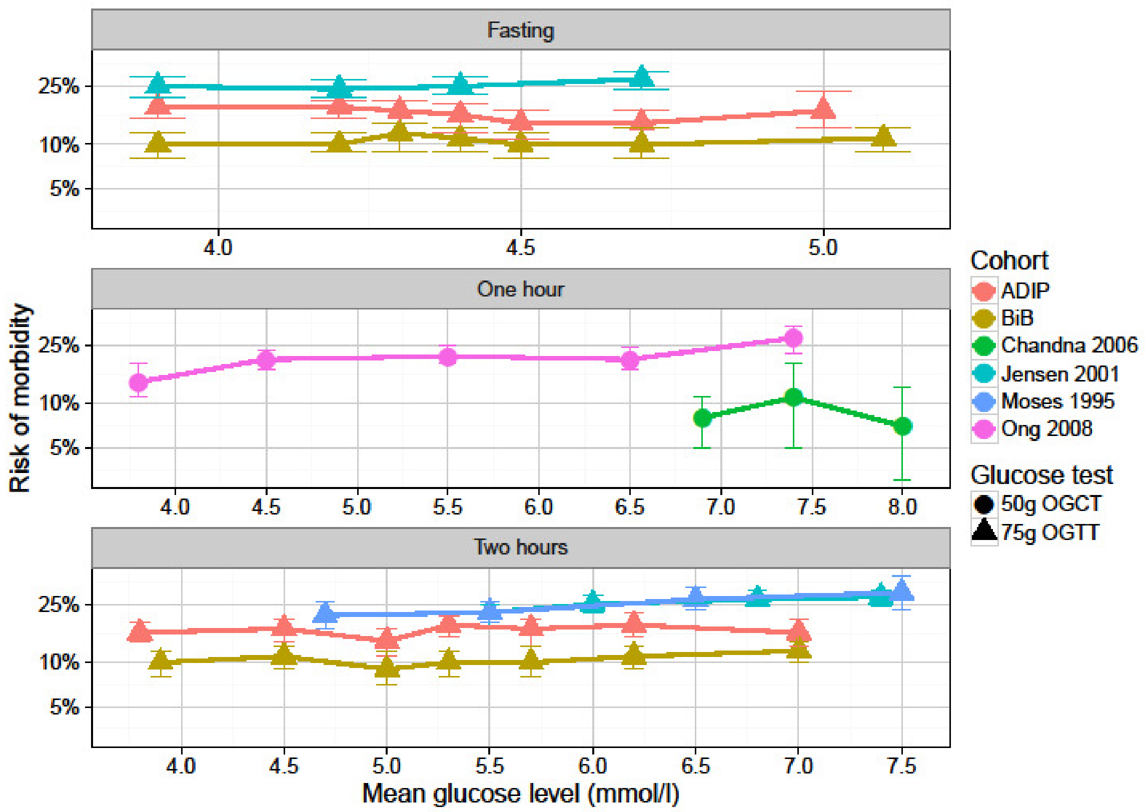

Fig F Risk of induction of labour against glucose concentration for each study, glucose test, and timing

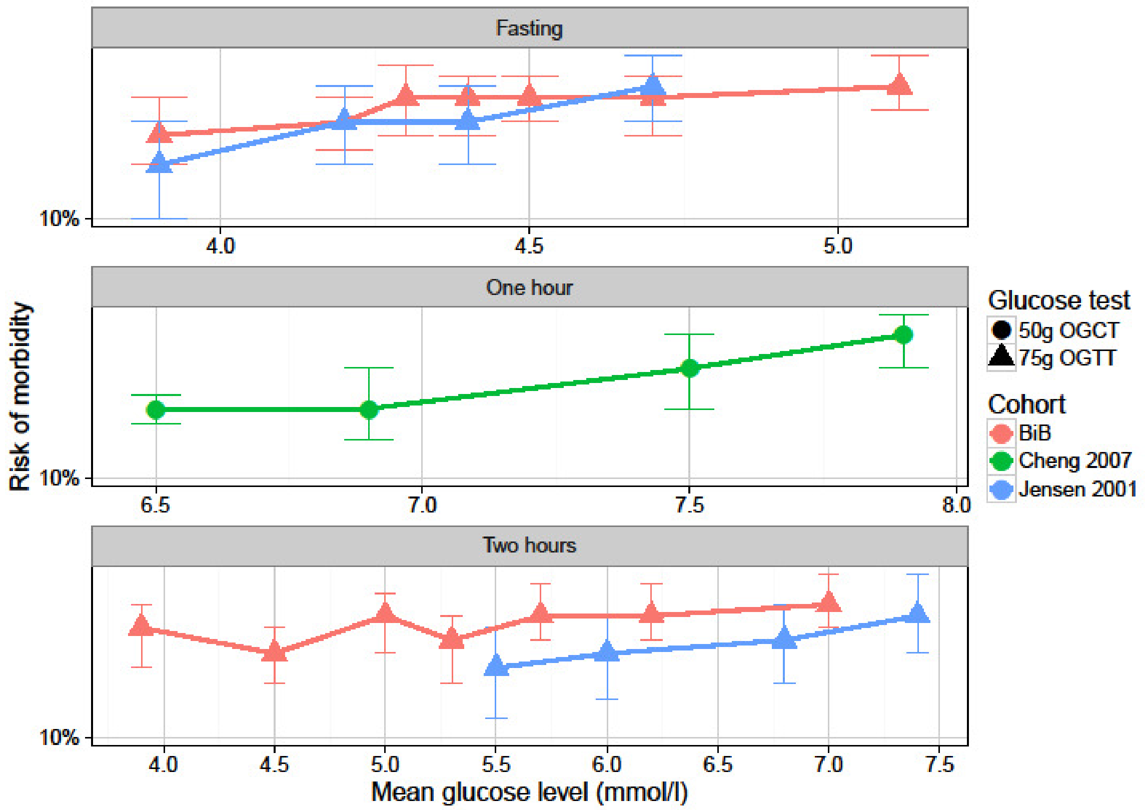

Fig G Risk of shoulder dystocia against glucose concentration for each study, glucose test, and timing

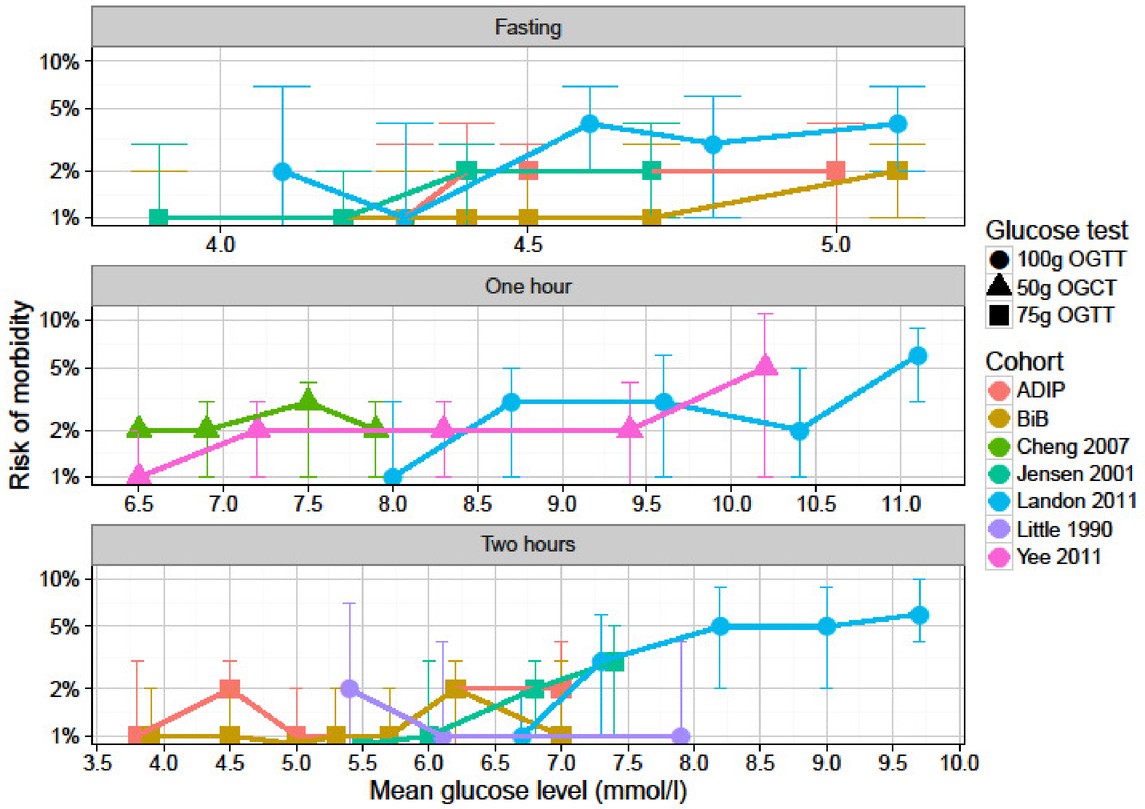

Fig H Risk of preterm birth against glucose concentration for each study, glucose test, and timing

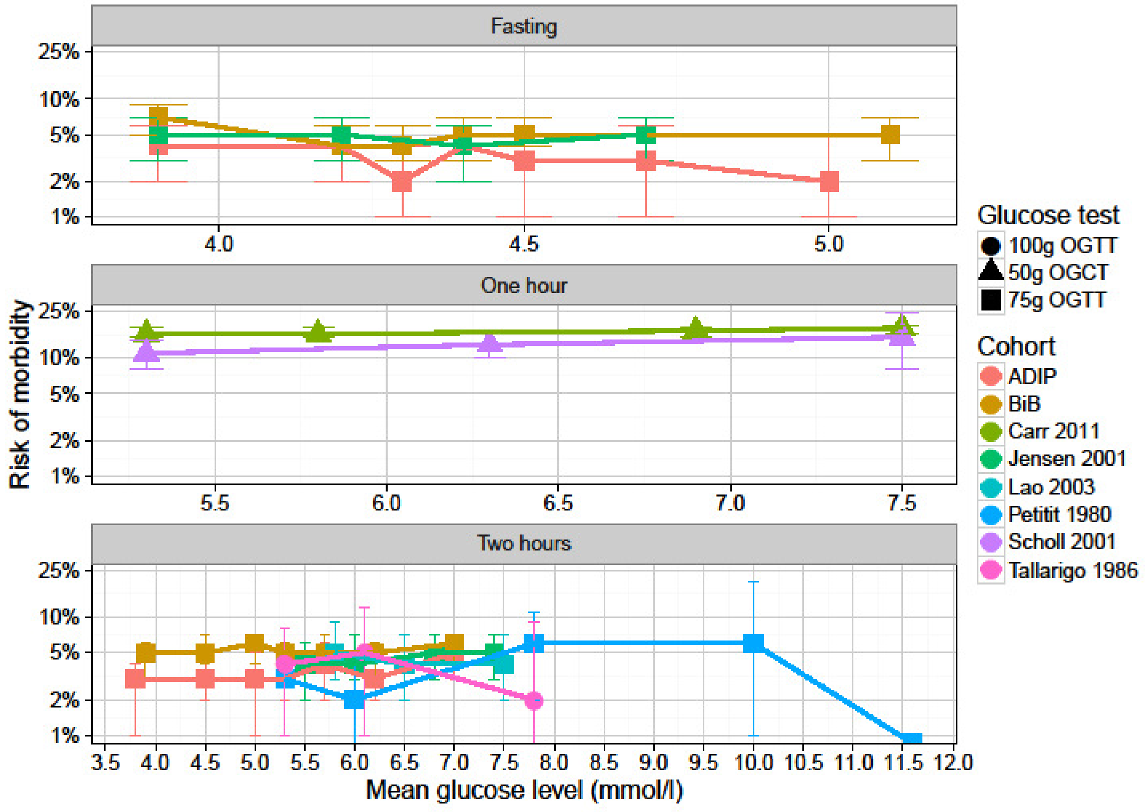

Fig I Risk of neonatal hypoglycaemia against glucose concentration for each study, glucose test, and timing

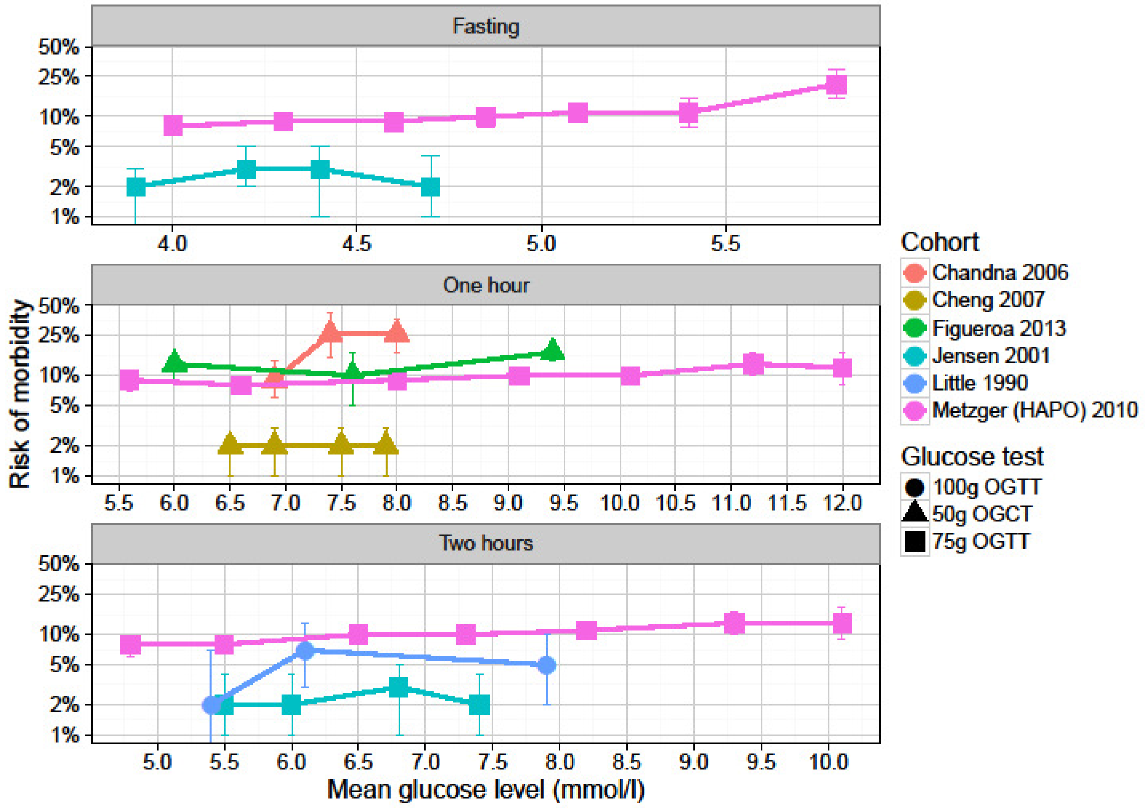

Supplement: Supplementary file 4 — Appendix 4: Risks of all outcomes against glucose concentration for each study, test, and timing [file fard032237.ww4_default.pdf]
